# SPP1^hi^ macrophages, NKG7 T cells, CCL5^hi^ fibroblasts, and IgM plasma cells are dominant features of necrobiosis

**DOI:** 10.1172/jci.insight.178766

**Published:** 2025-02-24

**Authors:** Stephanie T. Le, Alina I. Marusina, Alexander A. Merleev, Amanda Kirane, Olga Kruglinskaya, Andrey Kunitsyn, Nikolay Yu Kuzminykh, Xianying Xing, Sophie Y. Li, William Liakos, J. Michelle Kahlenberg, Andrea Gompers, Lauren Downing, Sahiti Marella, Allison C. Billi, Paul W. Harms, Lam C. Tsoi, Marie-Charlotte Brüggen, Iannis E. Adamopoulos, Johann E. Gudjonsson, Emanual Maverakis

**Affiliations:** 1 Department of Dermatology, and; 2 Department of Surgery, University of California, Davis, Sacramento, California, USA.; 3 Physioseq, Sacramento, California, USA.; 4 Institute of Biochemical Physics, Russian Academy of Science, Moscow, Russia.; 5 Department of Dermatology,; 6 Department of Internal Medicine, Division of Rheumatology,; 7 Department of Pathology, and; 8 Department of Computational Medicine and Bioinformatics, Department of Biostatistics, University of Michigan, Ann Arbor, Michigan, USA.; 9 Department of Dermatology, University Hospital Zurich, Zurich, Switzerland.; 10 Swiss Institute for Allergy Research, Davos, Switzerland.; 11 Division of Rheumatology and Clinical Immunology, Beth Israel Medical Deaconess Center, Boston, Massachusetts, USA.

**Keywords:** Dermatology, Immunology, Autoimmune diseases, Fibrosis, Skin

## Abstract

Necrobiosis is a histologic term used to describe abnormal deposits of “degenerating” collagen within the skin. It can be found as an incidental finding in various granulomatous conditions, but is a hallmark of necrobiosis lipoidica (NL) and necrobiotic xanthogranuloma (NXG). There is limited prior research on necrobiosis. Here, we employed single-cell analysis of lesional and nonlesional skin to study the pathophysiology of necrobiosis. Our findings demonstrate that necrobiotic lesional skin is characterized by SPP1^hi^ macrophages expressing *MARCO*; *NKG7*-expressing effector CD8^+^ T cells coexpressing *CCL5*, *IFNG*, *GZM*s, and *PRF1*; CCL5^hi^ fibroblasts coexpressing *CXCL9*, diverse collagens (e.g., *COL4A4*, *COL11A1*, *COL8A1*), and *TIMP1*; and *IGHM*-expressing plasma cells. Integrative analysis of signaling ligands and receptor expression identified strong cell-cell communication between NKG7^+^ T cells, CCL5^hi^ fibroblasts, and SPP1-expressing macrophages. In contrast, these cell populations were not dominant features of systemic sclerosis, another collagen deposition disease. Furthermore, although SPP1-expressing macrophages were detectable in sarcoidosis, *IFNG*-expressing T cells were a more defining feature of sarcoidosis compared with NL and NXG. From these findings, we speculate that necrobiosis results from the deposition of diverse collagens and ECM proteins through a process driven by CCL5-expressing fibroblasts and SPP1-expressing macrophages.

## Abstract 2

This study associates necrobiosis (altered collagen deposition) with increased expression of diverse extracellular matrix proteins and novel immune populations, especially SPP1-expressing macrophages and CCL5-expressing fibroblasts.

## Introduction

Necrobiosis is a term used to describe deposits of “degenerating” collagen fibers that are irregular in size and shape on histology. Other histologic descriptions of necrobiosis include “smudged,” “dead,” or “altered” collagen (1–3). Although necrobiosis can be seen in various granulomatous conditions, it is a hallmark of 2 diseases: necrobiosis lipoidica (NL) and necrobiotic xanthogranuloma (NXG). These are treatment-refractory, rare diseases that have similar histology but differ clinically, with NL most commonly occurring on the anterior lower extremities and NXG on the head, neck, trunk, and proximal extremities. NL and NXG also occur in different patient cohorts. NL is traditionally seen in diabetic patients, while NXG is most commonly seen in patients with monoclonal gammopathy (2).

The histories of the 2 diseases are also different. NL was first described in 1929 by Oppenheim as “dermatitis atrophicans diabetica” (4) and was subsequently renamed “necrobiosis lipoidica diabeticorum” in 1932 by Urbach (5). Since then, NL patients without a history of diabetes have been reported, prompting clinicians to drop “diabeticorum” from its name, shortening it to simply necrobiosis lipoidica (6). In contrast, NXG was first described much later by Kossard and Winkelmann, who, in 1980, recognized it as a periorbital dermatosis seen in patients with paraproteinemia or a myeloproliferative disorder (7).

Histologically, NL and NXG lesions are hallmarked by interstitial and palisading granulomas composed of non-epithelioid histiocytes, giant cells, lymphocytes, and plasma cells. The inflammatory infiltrate is separated by layers of necrobiotic collagen in a “wedding cake” pattern (NL) or an intersecting “X” pattern (NXG). Another histologic difference is that cholesterol clefts within the layers of necrobiosis are a more common feature of NXG. In both conditions, leukocytoclasia and fibrinoid necrosis in dermal blood vessels are not uncommon (1, 3).

To date, the pathophysiology of necrobiosis is poorly understood. Herein, we use bulk RNA sequencing (RNA-seq) and single-cell RNA-seq (scRNA-seq) of fresh tissue specimens, along with immunohistochemistry (IHC) and single-cell gene expression flex-fixed RNA profiling (FRP) on archived specimens, to characterize the gene expression and cellular profiles of necrobiosis. Our results demonstrate the cell types involved in NL pathophysiology, as well as unique gene expression profiles associated with both NL and NXG. Finally, we discuss therapeutic targets for these previously poorly characterized, treatment-refractory skin diseases.

## Results

### Similarities in NL and NXG gene expression.

**Figure 1:**
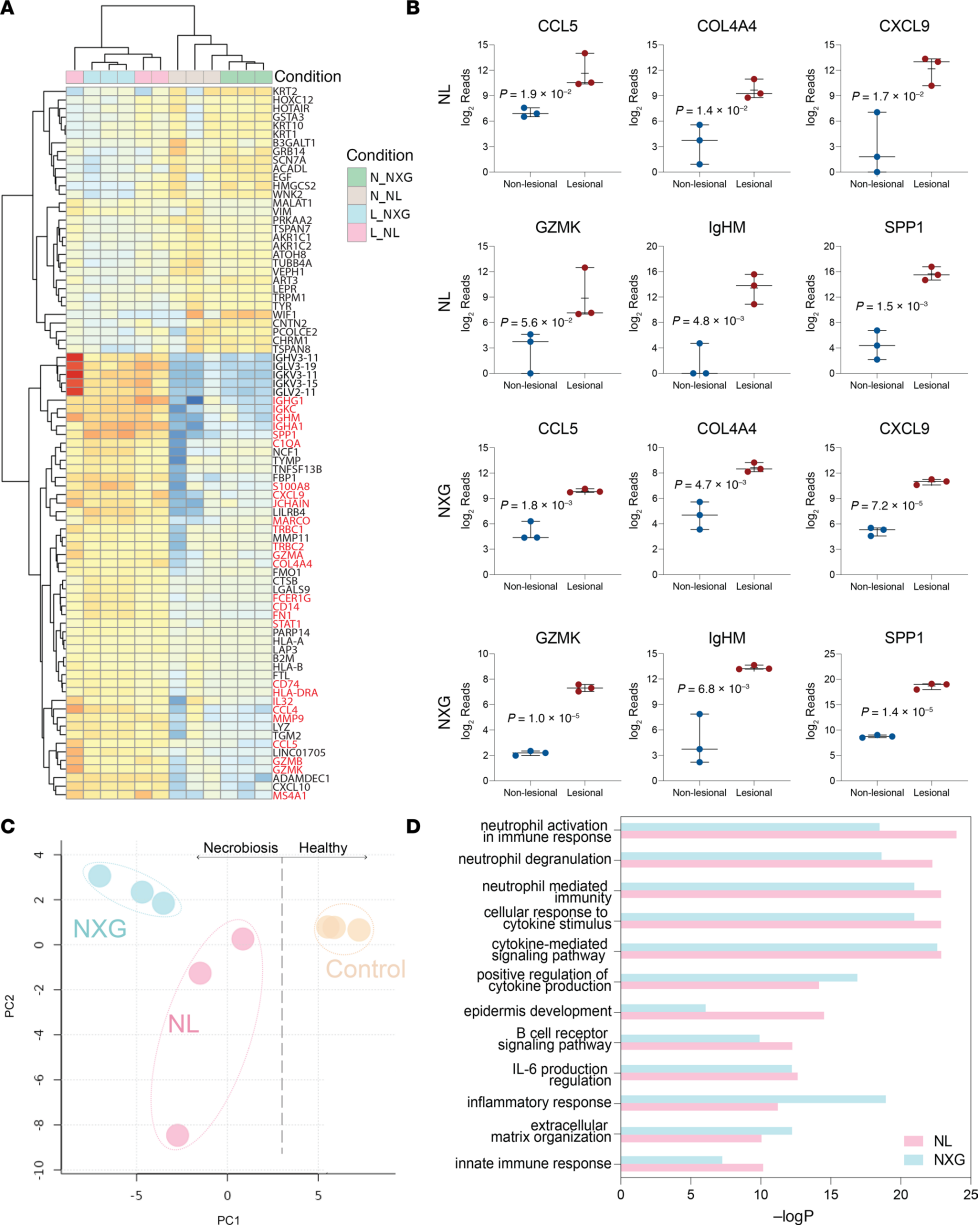
RNA-seq identifies *CCL5*, *COL4A4*, *CXCL9*, *SSP1*, and *IGHM* as consistently elevated in NL and NXG lesional skin. (**A**) Heatmap demonstrating hierarchical clustering of a select list of variably expressed genes separates NL and NXG lesional skin biopsy samples (pink and blue columns, respectively) from paired nonlesional skin samples. (**B**) Plots of individual differentially expressed genes. (**C**) Principal component analysis (PCA) of RNA-seq gene expression data comparing NL and NXG lesional skin to control healthy skin. (**D**) Gene expression pathway analysis of RNA-seq data identifies similar immune-related pathways as being differentially expressed in NL and NXG lesional skin.

Necrobiosis is a predominant histologic feature of both NL and NXG. To compare the gene expression profiles of these 2 diseases, RNA-seq was performed on lesional and nonlesional skin samples. Hierarchical clustering divided the samples into 2 major groups, clearly separating NL and NXG lesional from nonlesional samples (Figure 1A). Of the genes differentially expressed between paired lesion and nonlesion samples, strong differences were noted in B cell receptor (BCR) gene segments, T cell receptor gene (TCR) segments, *MS4A1* (CD20), *SPP1* (osteopontin [OSTP]), *C1QA* (complement), *FN1* (fibronectin), *CD74*, MHC class II genes (*HLA-DRA*), chemokines (*CCL5*, *CXCL9*, and *CXCL10*), granzymes (*GZMA*, *GZMB*, and *GZMK*), genes of macrophage differentiation (e.g., *MARCO*), and *COL4A4* (Figure 1, A and B). Principal component analysis (PCA) was performed to compare NL and NXG samples to healthy controls. The resulting PCA plot clearly separated NL and NXG lesional samples from healthy controls (Figure 1C, vertical axis), highlighting the similarity between NL and NXG gene expression.

**Figure 2:**
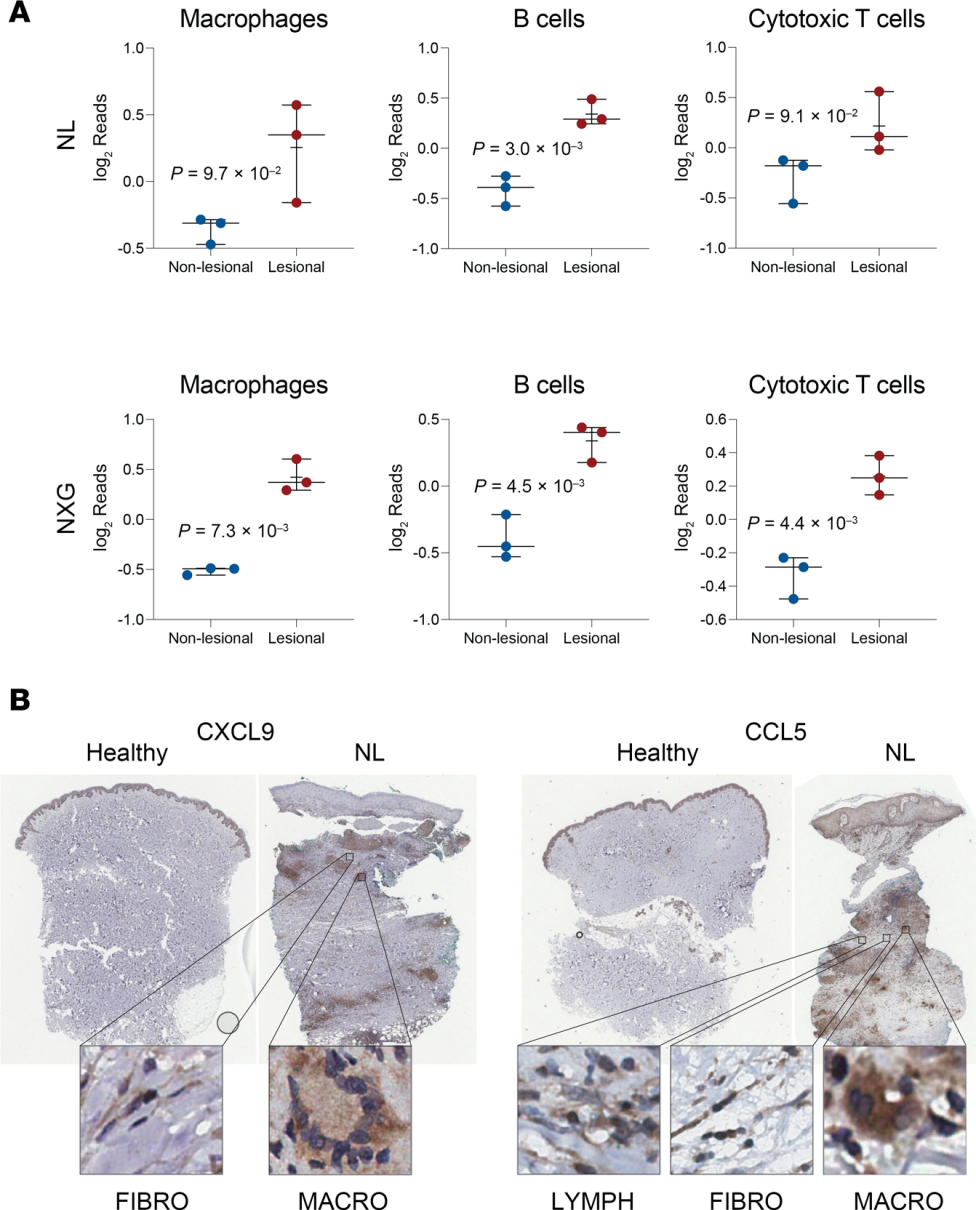
Deconvolution analysis identifies increased macrophages, B cells, and cytotoxic T cells in NL and NXG lesional skin. (**A**) CIBERSORT deconvolution analysis of RNA-seq data estimates cellular composition of NL and NXG lesional skin. (**B**) IHC analysis of CXCL9 and CCL5 expression in NL lesional and healthy control skin identifies high chemokine expression in NL macrophages, fibroblasts, and lymphocytes. Original magnification, ×40 (zoomed-in images).

To quantify immunocyte constituents from the bulk RNA-seq datasets, CIBERSORT deconvolution analysis was performed (8). While no increase in Th17 T cells was detected, CIBERSORT analysis revealed that lesional NL and NXG skin were associated with increased populations of macrophages, B cells, T cells, cytotoxic T cells, and neutrophils (Figure 2A and Supplemental Figure 1; supplemental material available online with this article; https://doi.org/10.1172/jci.insight.178766DS1). Pathway enrichment analysis (Figure 1D) revealed pathway changes in NL and NXG related to the putatively expanded cell types in lesional skin (Figure 2A and Supplemental Figure 1). Specifically, neutrophil activation in immune response, cellular response to cytokine stimulus, BCR signaling pathway, extracellular matrix (ECM) organization, and innate immune response were the pathways most significantly upregulated in lesional necrobiotic skin (Figure 1D).

### IHC of lesional skin identifies fibroblasts, T cells, and macrophages as major sources of differentially expressed cytokines in NL lesional skin.

Bulk RNA-seq identified differential expression of various cytokine genes in NL and NXG lesional skin (Figure 1, A and B). To verify these results at the protein level, IHC was performed. When compared with healthy skin, NL lesional skin was hallmarked by spindle-shaped cells (fibroblasts) expressing high levels of CCL5, CXCL9, and to a lesser extent, IL-32; large oval and multinucleated cells (macrophages) expressing high levels of CCL4, CCL5, CXCL9, and OSTP; and cells with spherical nuclei (lymphocytes) expressing high levels of CCL4, CCL5, and to a lesser extent, IL-32 (Figure 2B and Supplemental Figure 2). OSTP IHC was also performed on NL and NXG lesional skin and healthy control skin, which identified large oval and multinucleated cells (macrophages) expressing high levels of OSTP in NL and NXG lesional skin, with the expression of OSTP appearing greater in the latter (Supplemental Figure 3).

### NL lesional skin is associated with increased macrophages, T cells, and B cells.

**Figure 3:**
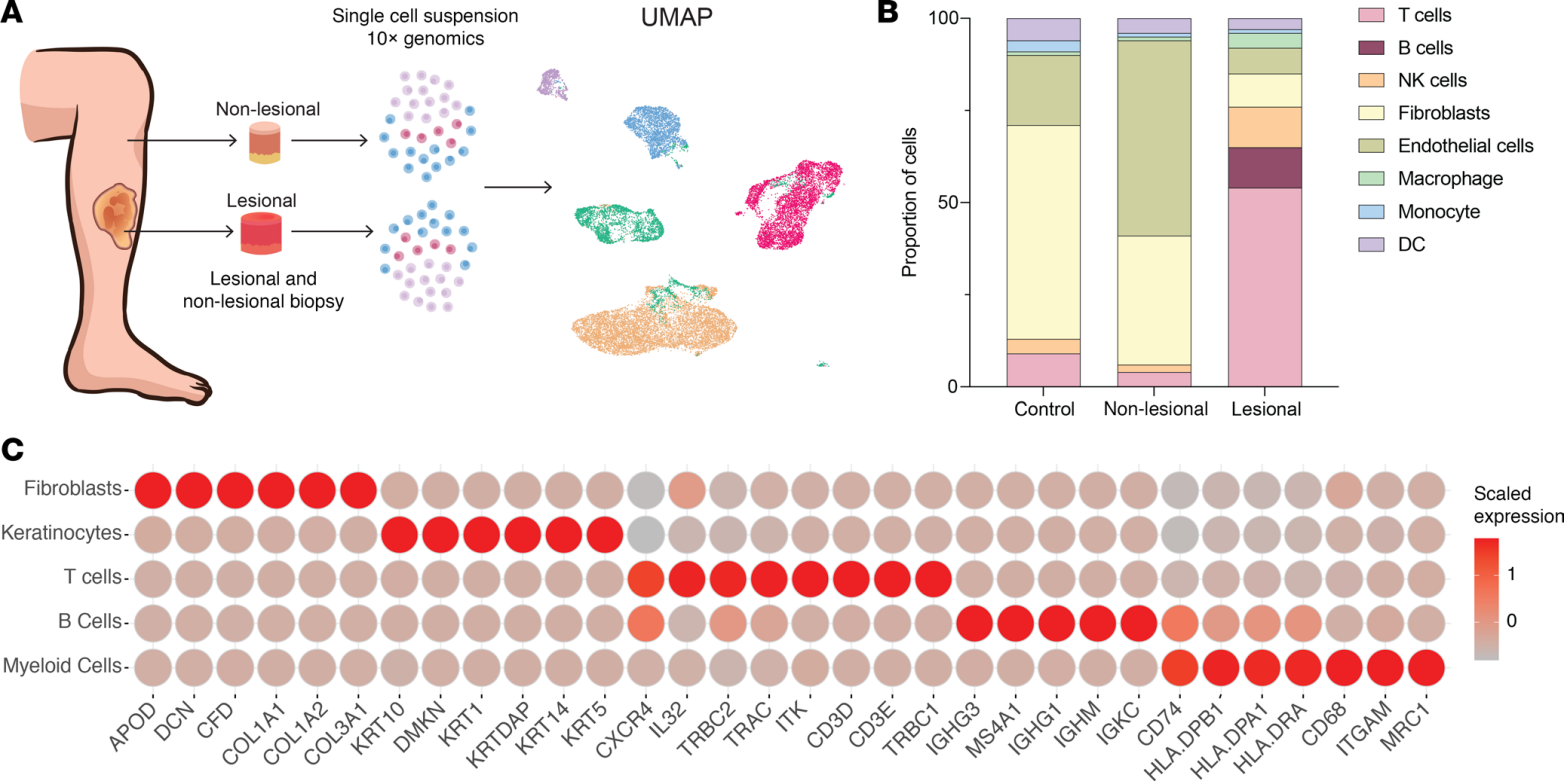
scRNA-seq of lesional and nonlesional NL skin samples. (**A**) Diagram of the experimental approach, depicting sites of lesional and nonlesional NL skin biopsies and 10× Genomics analysis. (**B**) Stacked bar chart showing cell proportions in healthy, NL nonlesional, and NL lesional skin. (**C**) Heatmap of marker genes used to define cell populations for subsequent analysis.

To obtain a better understanding of the cellular composition of NL, scRNA-seq was performed on fresh NL lesional and nonlesional skin samples, as well as a healthy control skin sample (Figure 3A). In total, 20,057 cells were sequenced, 19,532 of which were of mesenchymal or immune origin. Consistent with the deconvolution analysis of the bulk RNA-seq datasets (Figure 2A and Supplemental Figure 1), scRNA-seq revealed increased percentages of macrophages, T cells, and B cells in NL lesional skin (Figure 3B). Marker genes used to confirm cell type assignments (keratinocyte, myeloid, B cell, T cell, and fibroblast) are shown in Figure 3C.

### scRNA-seq identifies a CCL5-epxressing fibroblast population in NL lesional skin.

**Figure 4:**
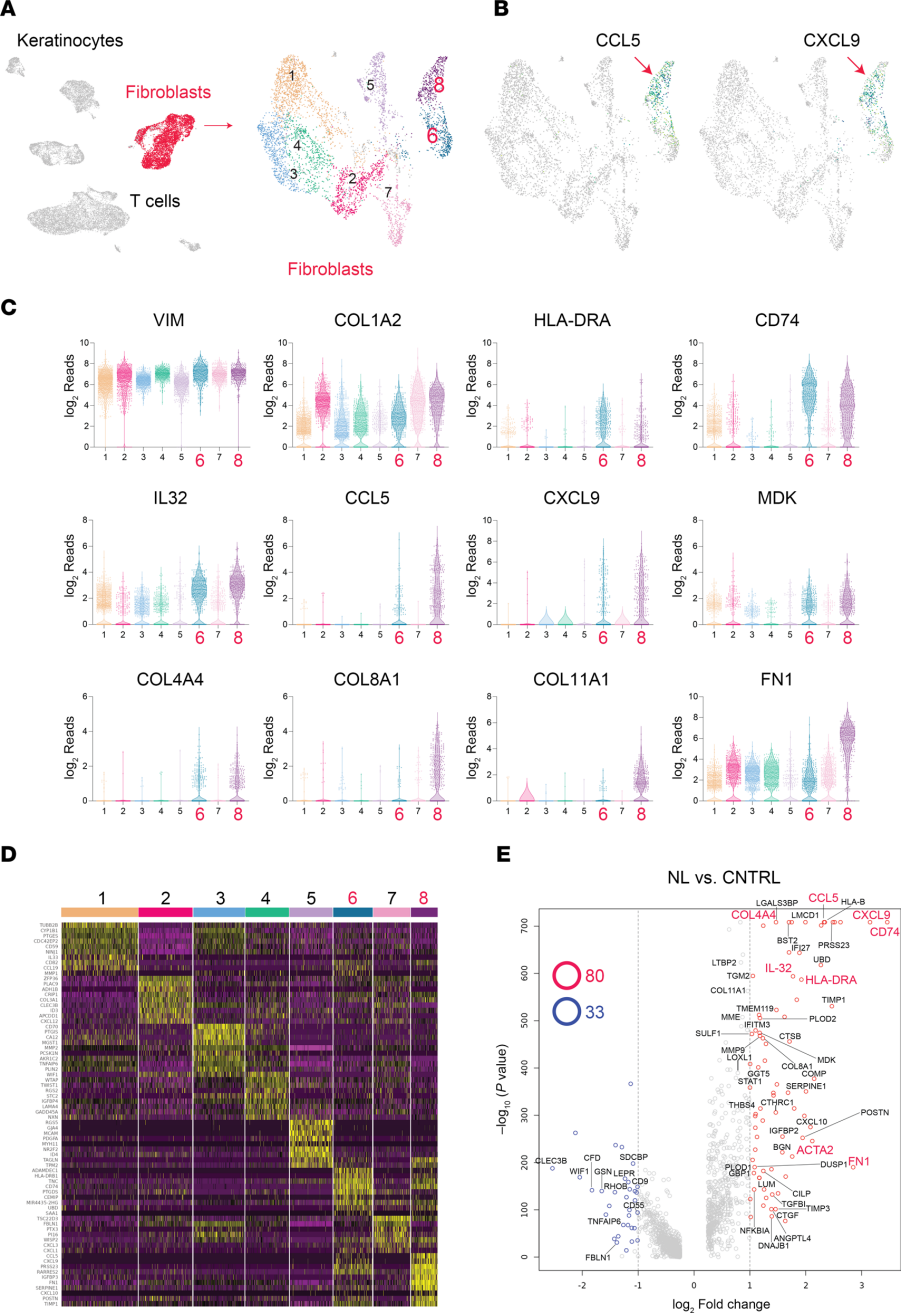
Single-cell analysis identifies a population of CCL5^hi^ fibroblasts in NL lesional skin. (**A**) Uniform manifold approximation and projection (UMAP) of NL lesional, nonlesional, and healthy control scRNA-seq data presents results as a 2-dimensional image. Each dot represents a single cell, and each cluster represents a group of cells with similar transcriptomes. A cluster with increased expression of mesenchymal genes was identified (red cluster labeled “Fibroblasts,” *n* = 5,041). Eight fibroblast subclusters were identified. (**B**) Distribution of *CCL5* and *CXCL9* expression across the 8 fibroblast clusters. The color range goes from yellow to dark blue, with dark blue indicating the highest expression. *CCL5* and *CXCL9* are predominantly expressed in Cluster 8 (red arrows), with less expression detected in Cluster 6. No or very little expression was detected in other clusters. (**C**) Violin plots of differentially expressed genes of interest across the 8 fibroblast clusters. (**D**) Heatmap of genes highly expressed in each of the 8 fibroblast subclusters. (**E**) Volcano plot comparing gene expression in NL lesional skin fibroblasts to control fibroblasts, identifying *COL4A4*, *CCL5*, *CXCL9*, *CD74*, and *HLA-DRA* as the most significant differentially expressed genes. Other differentially expressed genes of interest include *FN1*, *ACTA2*, *COL8A1*, *MMP9*, and *TIMP1*.

The uniform manifold approximation and projection (UMAP) method was used to visualize the NL lesional, nonlesional, and healthy control scRNA-seq data as a 2-dimensional image (Figure 4A). In the image, each dot represents a single cell, and each cluster represents a group of cells with similar transcriptomes. A cluster with increased expression of mesenchymal genes was identified (Figure 4A, red cluster labeled “Fibroblasts”). These cells (*n* = 5,041) were then reanalyzed independently of the other clusters using the UMAP method. This revealed that the mesenchymal cells could be further divided into 8 subclusters, corresponding to different fibroblast and fibroblast-like populations (Figure 4A). Gene expression analysis of each cluster and a heatmap of differentially expressed marker genes for each cluster are shown in Figure 4, B–D. Briefly, Clusters 1, 2, 3, and 7 were composed of fibroblasts expressing the following genes: *CD*59, *CD82*, and *IL33*; *ID3*, *COL3A1*, and *CXCL12*; *CD70* and *PCSK1N*; and *CXCL1* and *CXCL3*, respectively (Figure 4D). Cluster 5 and Cluster 8 were composed of pericytes (*MCAM*- and *RGS5*-expressing cells) and myofibroblasts (*ACTA2*-, *CTGF*-, and *PLOD2*^hi^-expressing cells), respectively (Figure 4D).

Clusters 6 and 8 were unique in that the cells were almost entirely from NL lesional skin. Consistent with IHC analysis, *CCL5* and *CXCL9* were almost exclusively expressed in these NL-specific fibroblast subpopulations (Clusters 6 and 8; Figure 4, C and D). Note the increased expression of ECM-associated genes (*POSTN*, *TNC*, *FN1*, *TIMP1*, and *PRSS23*), chemokines (*MDK*, *CXCL9*, *CCL5*, and *IL32*), and cell-surface markers (*CD74* and *HLA-DR*) in Clusters 6 and 8 (Figure 4, C and D). A volcano plot of differentially expressed genes in all NL fibroblasts versus healthy control fibroblasts is shown in Figure 4E. *COL4A4*, *CCL5*, *HLA-B*, *HLA-DRA*, *CD74*, *TIMP1*, *MMP9*, and *IL32* were among the most significantly upregulated genes in NL lesional skin fibroblasts. Violin plots for some of these genes are shown in Figure 4C, demonstrating their upregulation in NL-specific Clusters 6 and 8. Although their gene expression profiles overlapped greatly, Cluster 8 differed from Cluster 6 in that Cluster 8 expressed very high levels of ECM genes (*COL11A1*, *COL8A1*, and *FN1*) (Figure 4C) and myofibroblast-associated genes (*ACTA2*, *CTGF*, and *PLOD2*) (Supplemental Figure 4). Cluster 8 fibroblasts will be referred to as CCL5^hi^ fibroblasts for the remainder of this paper. Single-cell gene expression FRP is a 10× Genomics single-cell RNA-profiling technique that can be performed on archived FFPE samples. FRP yields results similar to those of scRNA-seq. Therefore, to validate the scRNA-seq results obtained with fresh NL tissue and to characterize NXG lesional mesenchymal and immune cells, we performed FRP on NL and NXG lesional skin and healthy skin as controls (NL, *n* = 8; NXG, *n* = 8; healthy skin, *n* = 4). FRP analysis verified the results obtained by scRNA-seq and demonstrated a similar set of upregulated genes in NXG fibroblasts (Supplemental Figure 5). FRP analysis also demonstrated that *COL4A1*, *COL4A3*, and *COL6A3* were upregulated in NL and NXG lesional fibroblasts (Supplemental Figure 5), expanding the number of differentially expressed collagen genes associated with necrobiosis. FRP analysis also identified additional proteinase-encoding genes upregulated in NL and NXG lesional fibroblasts, including *ADAM12*, *MMP11*, and *MME* (Supplemental Figure 5).

### CCL5 and CXCL9 are coexpressed in NL fibroblasts, but are induced by different stimuli.

Because high expression of *CCL5* and *CXCL9* was a hallmark of NL and NXG lesional fibroblasts, we next sought to determine whether the expression of these chemokine genes correlated with one another. Supplemental Figure 6A demonstrates that intracellular expression of these 2 genes highly correlated with one another in Cluster 8 fibroblasts (*r* = 0.6, *P* = 1.8 × 10^–36^). However, despite this strong intracellular correlation, the signals that induce cultured fibroblasts to express *CCL5* and *CXCL9* were very different (Supplemental Figure 6B). Specifically, the expression of *CCL5* was strongly induced by IFN-α, TNF, and IL-1β. To a lesser extent, IL-4, IL-13, IL-36, and TGF-β also stimulated fibroblasts to express *CCL5*. In contrast, IFN-α and IFN-γ were the only strong inducers of *CXCL9* (Supplemental Figure 6B). Importantly, in the absence of stimulation, both *CCL5* and *CXCL9* were not expressed at detectable levels by cultured fibroblasts (Supplemental Figure 6B), which corresponds to their negligible expression in healthy control skin fibroblasts (Figure 4C). In contrast, without stimulation, *IL32* was well expressed in cultured unstimulated fibroblasts (Supplemental Figure 6B), matching its baseline expression in healthy control skin fibroblasts (Figure 4C). *IL32* expression was upregulated further by stimulation with various cytokines, including IFN-α, IFN-γ, IL-13, IL-1β, IL-36, IL-4, TGF-β, and TNF (Supplemental Figure 6B).

### CCL5^hi^ fibroblasts are not a major subpopulation in systemic sclerosis and sarcoidosis.

To compare the results observed in NL to another disease with abnormal collagen deposition, we performed scRNA-seq on skin biopsy samples obtained from 4 patients with systemic sclerosis (aka scleroderma) and 3 healthy controls; a total of 37,337 mesenchymal and immune cells were sequenced. Similar to NL, a UMAP plot identified distinct fibroblast subpopulations (Supplemental Figure 6C). While none of the cell clusters were exclusively composed of systemic sclerosis fibroblasts, compared with other clusters, Clusters 1, 4, and 7 were composed of a high percentage of systemic sclerosis fibroblasts (69.2%, 74.2%, and 79.2%, respectively). Unlike NL-specific fibroblasts, these systemic sclerosis-enriched fibroblast clusters did not overexpress *IL32*, *CCL5*, or *CXCL9* (Supplemental Figure 6D). Furthermore, *COL4A4* and *COL8A1* were not meaningfully increased in these clusters (Supplemental Figure 6D). Next, we binned all systemic sclerosis fibroblasts into one group and then compared them to healthy control fibroblasts. This analysis revealed either poor expression or very modest increases in *MMP9*, *CCL5*, *CXCL9*, *IL32*, *CD74*, *COL4A4*, *COL8A1*, and *COL11A1* in systemic sclerosis fibroblasts, unlike their observed expression pattern in NL lesional CCL5^hi^ fibroblasts. However, systemic sclerosis fibroblasts did highly upregulate expression of *POSTN*, *TNC*, and *TIMP1* (Supplemental Figure 7A). Thus, there were some similarities between NL and systemic sclerosis fibroblasts, but an expansion of CCL5^hi^ fibroblasts appears to be unique to NL lesional skin. Also, the upregulation of *COL4A4*, *COL8A1*, and *COL11A1* in systemic sclerosis fibroblasts (Supplemental Figure 7A) was not nearly as strong as that observed in NL fibroblasts.

Although they are diseases of abnormal collagen deposition, NL and NXG are also considered to be noninfectious granulomatous diseases. Thus, sarcoidosis, a prototypical noninfectious granulomatous disease, was chosen as a second control for comparison. Reanalysis of a sarcoidosis scRNA-seq dataset revealed that, similar to NL and NXG, sarcoidosis lesional fibroblasts expressed high levels of MHC (e.g., *HLA-B* and *HLA-DRA*), *CD74*, *PLOD2*, *STAT1*, *TNC*, *PRSS23*, *PSMB9*, and *CXCL9* (Supplemental Figure 7B). However, unlike NL and NXG, *COL4A4*, *COL6A6*, and *COL11A1* were not significantly upregulated in sarcoidosis fibroblasts, and *CCL5* was only modestly upregulated (Supplemental Figure 7, B and C). Thus, while *CD74*- and *CXCL9*-expressing inflammatory fibroblasts are present in sarcoidosis, their phenotype does not perfectly match the CCL5^hi^ fibroblasts seen in NL and NXG fibroblasts.

### NL-specific macrophages express high levels of MARCO, SPP1, CCL4, and CCL5.

**Figure 5:**
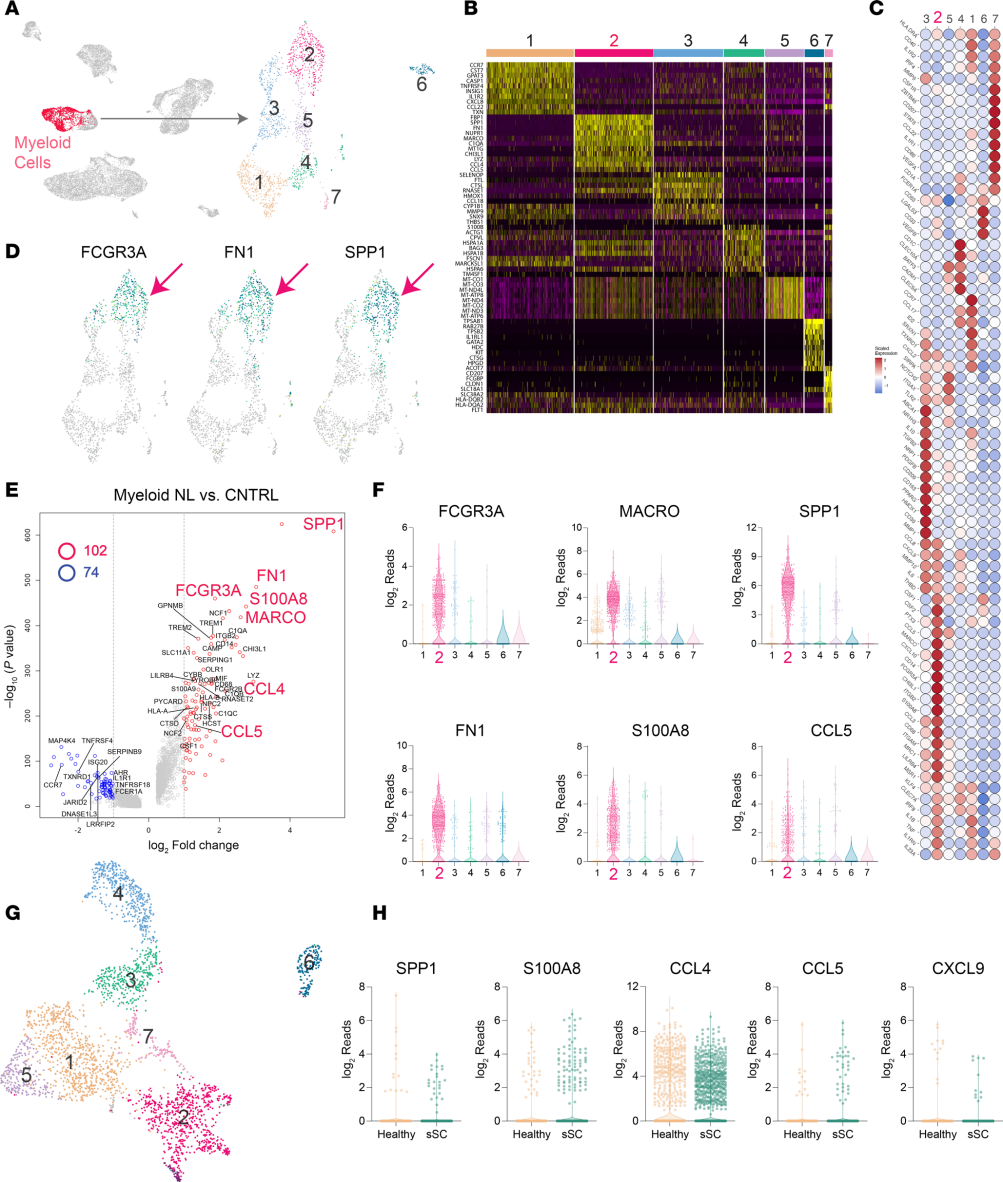
Single-cell analysis identifies a population of SPP1^hi^ macrophages in NL lesional skin. (**A**) The UMAP method was used to visualization of scRNA-seq data from NL lesional, nonlesional, and healthy control skin samples as a 2-dimensional image. On the right, each red dot represents a single myeloid cell (*n* = 1,757), which were then independently reanalyzed, identifying 7 myeloid subclusters. (**B**) Heatmap showing genes highly expressed in each of the 7 myeloid subclusters. (**C**) Heatmap of genes that are critical for identifying myeloid cell subtypes. (**D**) Distribution of *FCGR3A*, *FN1*, and *SPP1* expression across the 7 myeloid clusters. The color range goes from yellow to dark blue, with dark blue indicating the highest expression. *FCGR3A*, *FN1*, and *SPP1* are predominantly expressed in Cluster 2 (red arrows). No or very little expression was detected in other clusters. (**E**) Volcano plot comparing NL lesional skin myeloid cells to control myeloid cells, identifying *SPP1*, *FN1*, *FCGR3A*, *S100A8*, *MARCO*, *CCL4*, and *CCL5* as the most significant differentially expressed genes. (**F**) Violin plots showing the differential expression of genes of interest across the 7 myeloid clusters. (**G**) UMAP of systemic sclerosis (SS) scRNA-seq myeloid cell data from lesional skin and healthy control. (**H**) Violin plots demonstrating that *SPP1*, *S100A8*, *CCL4*, *CCL5*, and *CXCL9* are not strongly upregulated in systemic sclerosis (fold changes = 0.64, 1.5, 0.67, 1.73, and 0.27, respectively).

The UMAP method was used to visualize the NL lesional, nonlesional, and healthy control scRNA-seq data as a 2-dimensional image (Figure 5A). A cluster with increased expression of myeloid genes was identified (Figure 5A, red cluster labeled “Myeloid Cells”). These cells (*n* = 1,757) were then reanalyzed independently using the UMAP method. This revealed that the myeloid cells could be further divided into 7 distinct subclusters (Figure 5A, right side). Of these, Cluster 2 was unique in that it was derived solely from NL lesional myeloid cells.

Figure 5B is a heatmap of differentially expressed marker genes for each cluster, and Figure 5C is a heatmap of genes critical for subtyping myeloid cells. Cluster 1 was composed of activated dendritic cells (DCs) expressing high levels of cell surface markers *CD1C*, *HLA-DR*, *CD40*, and *CCR7*; transcription factors *ID2*, *IRF4*, and *IRF8*; and various inflammatory cytokines, e.g., *IL1B*, *TNF*, *CCL17*, and *IL23A*. Cluster 2 was unique in that it was solely composed of NL lesional macrophages, i.e., there were no Cluster 2 cell contributions from healthy and nonlesional skin. Cluster 2 was composed of macrophages that were notable for their very high expression of *SPP1* (the gene that encodes OSTP), *MARCO*, *FN1*, *C1QA*, *CCL4*, *CCL5*, and *S100A8*. Cluster 3 was also composed of macrophages, but compared with Cluster 2, they expressed low levels of *SPP1*, *MARCO*, *FN1*, *CCL5*, and *S100A8* (Figure 5, B–D). Cluster 4 contained mainly conventional type 1 DCs, as evident by their increased expression of *BTLA*, *CADM1*, *CLEC9A*, *LY75*, *XCR1*, and *ID2* (Figure 5C). Cluster 5 was composed of stressed cells (elevation of numerous mitochondrially encoded genes), possibly a result of the overnight single-cell isolation process or the NL disease state itself. Cluster 6 was composed of mast cells and/or basophils (*CD63*^hi^, *KIT*, *FCER1A*, *CMA1*, *TPSAB1*, and *TPSB2* [which encode tryptase], and *HDC*), and finally, Cluster 7 was composed of *CD207*-expressing Langerhans cells (Figure 5C).

Differential gene expression analysis of all NL lesional skin myeloid cells compared with all myeloid cells present in nonlesional and healthy skin was performed, and results are displayed as a volcano plot (Figure 5E). There were 102 upregulated and 74 downregulated genes. *SPP1*, *FN1*, *FCGR3A*, *S100A8*, *CCL4*, and *MARCO* were among the most significant upregulated differentially expressed genes in NL lesional skin. These genes were also found to be upregulated in NL and NXG by bulk RNA-seq, in lesional versus nonlesional skin samples (Figure 1A), and they were the most highly upregulated genes in NL-specific myeloid cell Cluster 2 (Figure 5, D and F, and Supplemental Figure 8A). In the remainder of the text, we will refer to Cluster 2 cells as *SPP1*^hi^ macrophages.

Although CXCL9 was shown by IHC to be strongly upregulated in NL lesional macrophages, *CXCL9* was not exclusively expressed in Cluster 2 myeloid cells. To investigate this further, macrophages were binned into 2 groups, NL lesional macrophages and control macrophages (healthy skin macrophages and nonlesional macrophages). Expression of *CXCL9* was exclusively observed in NL lesional macrophages (Supplemental Figure 8B), which matched the CXCL9 IHC data (Figure 2B). This comparison (NL lesional skin versus nonlesional and healthy skin) revealed additional differentially expressed genes in NL lesional macrophages, including a modest increase in genes encoding M1 effector cytokines (*IL1A*, *IL1B*, and *IL6*) and a modest decrease in the M2 effector gene, *IL10* (Supplemental Figure 8B). Thus, at least in terms of genes encoding effector cytokines, NL lesional skin was associated with an M1 macrophage response. However, ultimately these changes were small in comparison with the upregulation of *SPP1* in NL lesional macrophages (Supplemental Figure 8B).

To validate the scRNA-seq results and to characterize NXG lesional macrophages, we performed FRP on NL and NXG lesional skin and healthy skin as control (Supplemental Figure 9). Genes found to be upregulated in NL lesional macrophages by scRNA-seq were also detected to be upregulated in NL and NXG lesional macrophages by FRP. In addition, FRP analysis identified several additional upregulated genes, including genes encoding cell surface receptors, *LGALS9* and *CXCR4*; and genes involved in macrophage effector function or polarization, *INHBA*, *DXML2*, and *IL32* (Supplemental Figure 9).

### SPP1^hi^ macrophages coexpressing CCL4 and CCL5 are not a dominant feature in systemic sclerosis or sarcoidosis.

SPP1 has been associated with lung fibrosis in systemic sclerosis. To determine whether the NL-specific elevations in *SPP1* and *CCL4* were also a feature of lesional systemic sclerosis skin–derived myeloid cells, we performed scRNA-seq on skin biopsy samples obtained from 4 patients with systemic sclerosis (aka scleroderma) and 3 healthy controls (in total, 2,777 myeloid cells were sequenced). The UMAP method was used to visualize the systemic sclerosis lesional and healthy control scRNA-seq data as a 2-dimensional image (Figure 5G). Similar to NL, 7 distinct populations of myeloid cells were observed. But unlike NL, the scRNA-seq analysis did not identify a systemic sclerosis–specific subpopulation of myeloid cells, i.e., myeloid cells derived from normal healthy skin were present in all 7 identified myeloid cell clusters. Thus, to compare the expression of *CCL4* and *SPP1* in systemic sclerosis myeloid cells versus healthy skin myeloid cells, scRNA-seq data from all systemic sclerosis myeloid cells were binned together and compared to all healthy skin–derived myeloid cells. This analysis revealed that there was no significant and meaningful (fold change [FC] > 2) upregulation in the expression of *SPP1*, *CCL4*, *CCL5*, and *CXCL9* in systemic sclerosis myeloid cells (Figure 5H). In fact, there was a decrease in *CCL4* expression (FC = 0.38) in systemic sclerosis–derived myeloid cells (Figure 5H), the opposite pattern to that observed in NL (Figure 5, B and E, and Supplemental Figure 8).

Sarcoidosis was chosen as an additional control to compare NL and NXG macrophages to lesional macrophages of another noninfectious granulomatous disease. Similar to NL and NXG, sarcoidosis lesional macrophages upregulated expression of the macrophage polarization marker genes *MARCO*, *FCGR3A*, *NR1H3*; the ECM-encoding gene *FN1*; the protease-encoding gene *CTSS*; and genes associated with inflammation, *S100A8* and *CHI3L1* (Supplemental Figure 10A). However, upregulation of *SPP1* was more modest, and unlike NL and NXG macrophages, sarcoidosis lesional macrophages strongly overexpressed *APOE*, *VAT1*, *TLR8*, and *ALDH1A1* among other genes (Supplemental Figure 10B).

### scRNA-seq reveals highly expanded NKG7^+^, CCL5^+^, and CD8A^+^ T cells in NL lesional skin.

**Figure 6:**
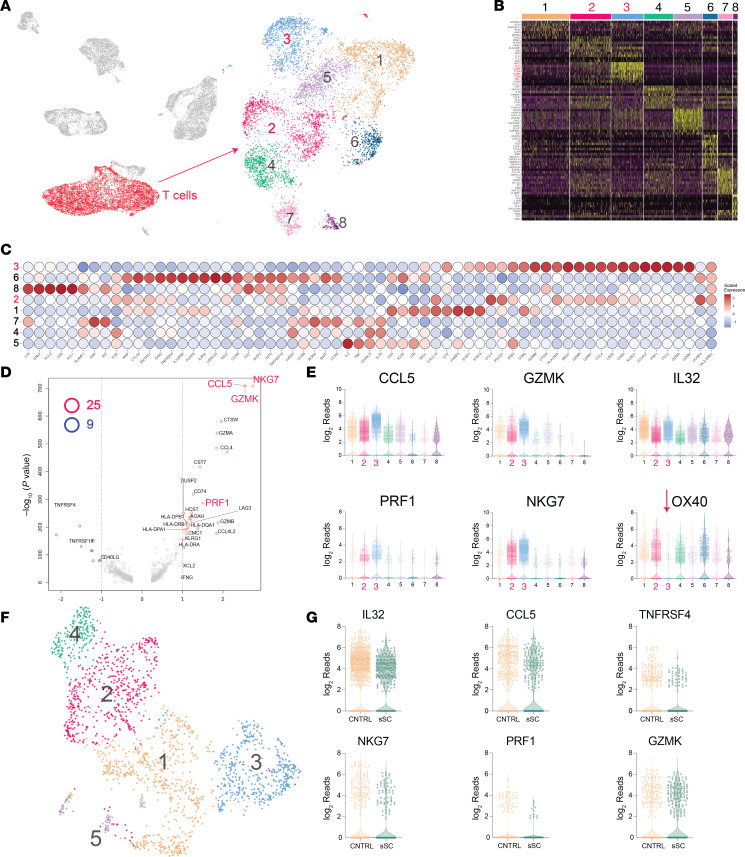
Single-cell analysis identifies a population of NKG7 T cells in NL lesional skin. (**A**) Left: UMAP 2-dimensional image visualization of scRNA-seq data from NL lesional, nonlesional, and healthy control skin samples. Each red dot represents a single T cell (*n* = 6,157). Right: When analyzed independently, 8 T cell subclusters were identified. (**B**) Heatmap showing genes highly expressed in each of the 8 T cell subclusters. (**C**) Heatmap of genes that are critical for identifying T cell subtypes. (**D**) Volcano plot comparing NL lesional skin T cells to control T cells, identifying *CCL5*, *NKG7*, *GZMK*, and *PRF1* as the most significantly differentially expressed genes. (**E**) Violin plots showing the differential expression of genes of interest across the 8 T cell clusters. (**F**) UMAP of systemic sclerosis (SS) T cell scRNA-seq data from lesional skin and healthy controls. (**G**) Violin plots demonstrating that *NKG7*, *CCL5*, and *PRF1* are not strongly upregulated in systemic sclerosis lesional T cells.

A UMAP of NL lesional, nonlesional, and healthy control scRNA-seq data is shown in Figure 6A, with a cluster corresponding to T cells highlighted in red. These cells (6,157 in total) were then reanalyzed independently of the other clusters using the UMAP method (Figure 6A, right side). This revealed that the T cell cluster could be further subdivided into 8 subclusters, corresponding to different T cell subsets. Cluster 2 and Cluster 3 were unique in that they were derived solely from NL lesional T cells. However, except for Clusters 6, 7, and 8, most T cell clusters were composed predominantly of NL lesional T cells, as NL lesional skin had a strong expansion of all T cell subsets (Figure 3B). Figure 6B is a heatmap of differentially expressed marker genes for each cluster, and Figure 6C is a heatmap of genes critical for classifying T cell subsets. Briefly, Cluster 1 was composed of CD4^+^ *IL32*-expressing recently activated T cells, which were characterized by high expression of *CCR7*, *SELL* (*CD62L*), *STAT1*, *STAT4*, and *CXCL13*. They also expressed *HLA-DR* and low to moderate levels of *CD25* and *FOXP3*, consistent with an activated phenotype. *GATA3* and *LY9* expression was very low in this cluster. NL-specific T cell clusters, Clusters 2 and 3, were composed of CD8^+^ T cells with medium and high expression of *NKG7*, respectively. Cluster 3 also expressed high levels of *HLA-DRA*, *IFNG*, and *CD84*, as well as genes encoding cytotoxic effector molecules, e.g., all *GZM*s (*GZMA*, -*B*, -*H*, and -*K*), and *PRF1*. Finally, like NL-specific macrophages, Cluster 3 T cells expressed high levels of *CCL4* and *CCL5*. Cluster 4 was composed of *TGFB1*-expressing, *GATA3*^hi^, *RORA*^hi^ T cells that expressed low levels of *STAT1* and the *STAT1*-associated cytokine *IFNG*. Cluster 5 T cells expressed *CD4*, *CD40LG*, *IL7R*, *LTB*, *STAT1*, and the Th1 cytokines *IL2*, *TNF*, and *IFNG*. Cluster 6 was composed of T regulatory cells, characterized by very high expression of *FOXP3*, *IL2RA* (the gene that encodes CD25), *TIGIT*, *ICOS*, *CCR8*, *CTLA4*, *GITR*, and *BATF*. Clusters 7 and 8 were composed of memory T cells. Both clusters expressed high *CXCR3*, *IL7R*, and *IL32*, and low *CCR7*, *HLA-DRA*, and *SELL* (the gene that encodes CD62L). However, these clusters differed in that Cluster 7 had high and Cluster 8 had low expression of *CCR4*, *TGFBR3*, and *RORA*. In contrast, Cluster 7 had low and Cluster 8 had high expression of *XCL1*, *XCL2*, *CTSW*, *AOAH*, *CD94*, and *GNLY* (Figure 6C).

A volcano plot of differentially expressed genes in NL lesional T cells versus healthy and nonlesional T cells is shown in Figure 6D. *NKG7*, *CCL5*, and *GZMK* were among the most significant upregulated genes in NL lesional skin (Figure 6D). These genes were expressed at highest levels in the NL-specific Cluster 3, highlighting the importance of this cluster. Violin plots of differentially expressed genes of particular interest are shown in Figure 6E. NL-specific Cluster 3 was noted to have the highest expression of *CCL5*, *GZMK*, *NKG7*, and *PRF1*, and the lowest expression of *OX40*. *IL32* was well expressed in all T cell clusters, but highest in Clusters 1 and 2. For the remainder of the paper, Cluster 7 T cells are referred to as IL-7^hi^ memory T cells, Cluster 8 T cells are referred to as XCL1 memory T cells, and Cluster 3 T cells are referred to as NKG7 T cells.

To validate the scRNA-seq results and to characterize NXG lesional T cells, we performed FRP on NL and NXG lesional skin and healthy skin as controls. Results revealed an increased percentage of *SELL*^+^ and *CCR7*^+^ T cells in NL and NXG (Supplemental Figure 11, A and B). There was also an upregulation of *GZMK* and *JAK3* in NL and NXG T cells and a significant increase in the expression of the Th1 transcription factor *STAT1* in NL and NXG T cells. Finally, there were some modest differences between the FRP and scRNA-seq T cell characterizations. Specifically, unlike the scRNA-seq results, FRP detected only a small upregulation of *CCL5* expression in NL and NXG T cells versus healthy control T cells (Supplemental Figure 11, A and B). However, when *NKG7*^+^ T cells were binned together and analyzed independently of other T cell populations, a strong, significant upregulation of *NKG7*, *GZMK*, and *CCL5* was noted in NL compared with healthy controls (Supplemental Figure 11C).

### NKG7- and CCL5-expressing T cells are not a dominant feature in systemic sclerosis or sarcoidosis.

For comparison to NL, scRNA-seq analysis of systemic sclerosis and control skin T cells was performed, as described above. The UMAP method was used to visualize the systemic sclerosis lesional and healthy control scRNA-seq data as a 2-dimensional image (Figure 6F; 1,867 total T cells). Five distinct subpopulations of T cells were observed; however, none were predominantly composed of systemic sclerosis T cells. T cells derived from healthy skin were present in all 5 identified T cell clusters. Thus, to compare the expression of *NKG7* and *CCL5* in systemic sclerosis versus healthy skin, systemic sclerosis T cells were binned together and compared as a group to healthy skin–derived T cells. This analysis revealed that there was no meaningful difference in the *NKG7* or *CCL5* expression in systemic sclerosis T cells versus healthy T cells; in fact, their expression was slightly lower (Figure 6G).

Sarcoidosis was again chosen as an additional control. Reanalysis of a sarcoidosis scRNA-seq dataset revealed some similarities between sarcoidosis lesional T cells and those of NL. Specifically, when compared with healthy control T cells, there was a modest upregulation of *NKG7* and *CCL5* in sarcoidosis lesional T cells (Supplemental Figure 12). However, the most striking feature of sarcoidosis lesional T cells was their very strong and significant upregulation of *STAT1* and *IFNG* (Supplemental Figure 12).

### IGMH-expressing B cells in NL and NXG.

**Figure 7:**
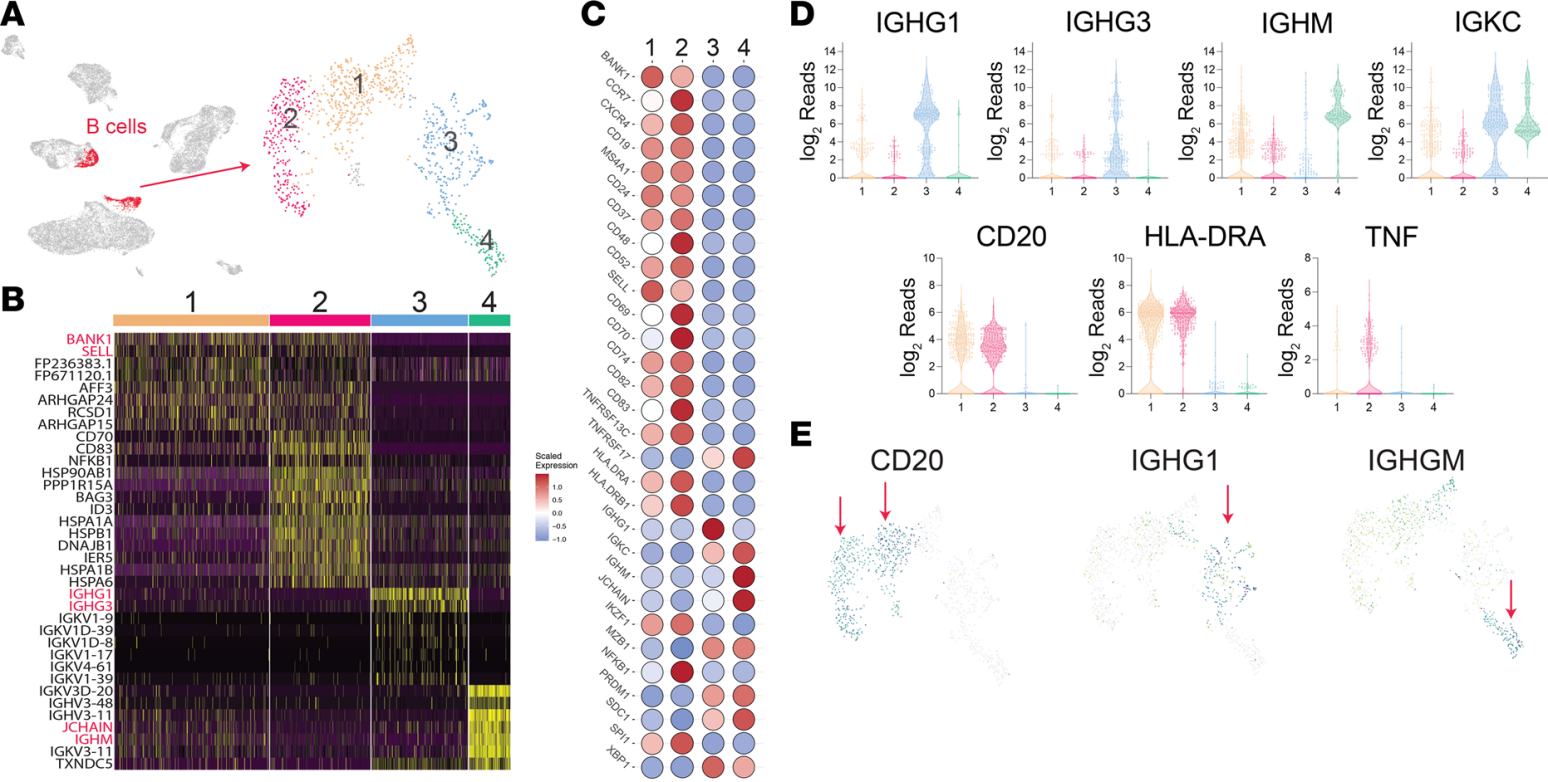
Single-cell analysis identifies a population of IGHM^+^ plasma cells in NL lesional skin. (**A**) UMAP visualization of scRNA-seq data from NL lesional, nonlesional, and healthy control skin samples. Each red dot represents 1 B cell (*n* = 1,162). When analyzed independently, 4 B cell subclusters were identified. (**B**) Heatmap showing genes highly expressed in each of the 4 B cell subclusters. (**C**) Heatmap of genes that are critical for identifying B cell subtypes. (**D**) Violin plots of differentially expressed genes of interest across the 4 B cell clusters. Note that *CD20* is not expressed in Clusters 3 and 4, while *IGHM* is highly expressed in Cluster 4. (**E**) Distribution of *CD20*, *IGHG1*, and *IGHM* expression across the 4 B cell clusters. The color range goes from yellow to dark blue, with dark blue indicating the highest expression. Note that *CD20* is only expressed in Clusters 1 and 2 (red arrows), while *IGHM* is highly expressed in Cluster 4 (red arrow).

B cells are not a major immune population in healthy skin; however, their frequency is elevated in many dermatologic conditions, including NL (Figure 3B). A UMAP of NL lesional, nonlesional, and healthy control scRNA-seq data is shown in Figure 7A, with 2 clusters corresponding to B cells highlighted in red. These cells (*n* = 1,162) were then reanalyzed independently of the other clusters using the UMAP method, which yielded 4 distinct B cell clusters (Figure 7A, right side). All clusters were composed of B cells derived solely from NL lesional skin, as there was only a single B cell detected in the control samples.

Figure 7B is a heatmap of differentially expressed marker genes for each cluster, and Figure 7C is a heatmap of genes critical for identifying B cell subsets. Briefly, Clusters 1 and 2 were composed of *MS4A1*-expressing activated memory B cells, and Clusters 3 and 4 were composed of plasma cells, i.e., *MS4A1*^–^ cells expressing characteristic plasma cell markers *PRDM1* (encodes BLIMP1), *SDC1* (encodes CD138), and/or *TNFRSF17* (encodes CD269). However, plasma cell Clusters 3 and 4 differed from one another in the class of Ig heavy chain they expressed, *IGHG1* and *IGHM*, respectively (Figure 7, D and E). Although isotype switching usually occurs before plasma cell differentiation, 40% of the NL *IGHM*-expressing B cells were plasma cells, as was evident by their lack of *MS4A1* expression and their expression of at least one plasma cell marker. Venn diagrams illustrating the percentage expression of each plasma cell marker, *PRDM1* (23.6%), *SDC1* (22.7%), and *TNFRSF17* (29.5%), are displayed in Supplemental Figure 13A. For the remainder of this paper, Cluster 4 cells will be referred to as IGHM-expressing plasma cells.

To validate the scRNA-seq results and to characterize NL and NXG lesional B cells, we performed FRP on NL and NXG lesional skin and healthy skin as controls. Similar to scRNA-seq results, FRP analysis confirmed that IGHM-expressing plasma cells were abundant in NL and NXG lesional skin (Supplemental Figure 13B). Although FRP captures a smaller proportion of the transcriptome in comparison with scRNA-seq, FRP analysis could clearly identify that many of the NL and NXG lesional B cells were indeed plasma cells. The precise ratio of IGHG- versus IGHM-expressing plasma cells is difficult to assess by FRP, but from the scRNA-seq data, it appears that in NL lesional skin, IGHG1-expressing plasma cells are slightly more abundant than IGHM-expressing plasma cells (IGG1 > IGM > IGG3; Supplemental Figure 13C).

Because IGHM-expressing plasma cells are not a well-recognized B cell subset, we investigated whether they could be detected in systemic sclerosis, another disease with pathogenic B cells and abnormal collagen deposition. Using the same systemic sclerosis scRNA-seq samples described previously, individual B cells (*n* = 229) were identified by their expression of classic B cell markers. From this population, B cells expressing the IgM heavy chain gene segment (IGHM) were identified (*n* = 153). The percentage of systemic sclerosis IGHM B cells expressing different B cell differentiation markers is displayed as Venn diagrams in Supplemental Figure 13A. Unlike NL, none of the lesional skin systemic sclerosis IGHM B cells expressed any of the plasma cell markers, *PRDM1* (BLIMP1), *SDC1* (CD138), or *TNFRSF17* (CD269) (Supplemental Figure 13A). The vast majority (92.8%) of the systemic sclerosis IGHM-expressing B cells expressed *MS4A1* (CD20), which is the typical expression pattern for IGHM-expressing B cells. As an additional control, a sarcoidosis dataset was also examined. In contrast with NL, B cells were minimally present in sarcoidosis, and virtually none of the detected B cells were plasma cells (Supplemental Figure 13D). Thus, it appears that IGHM-expressing plasma cells are somewhat unique to NL.

### scBCR and scTCR repertoire analysis identifies clonal expansions of IgM-expressing plasma cells and NKG7 T cells.

**Figure 8:**
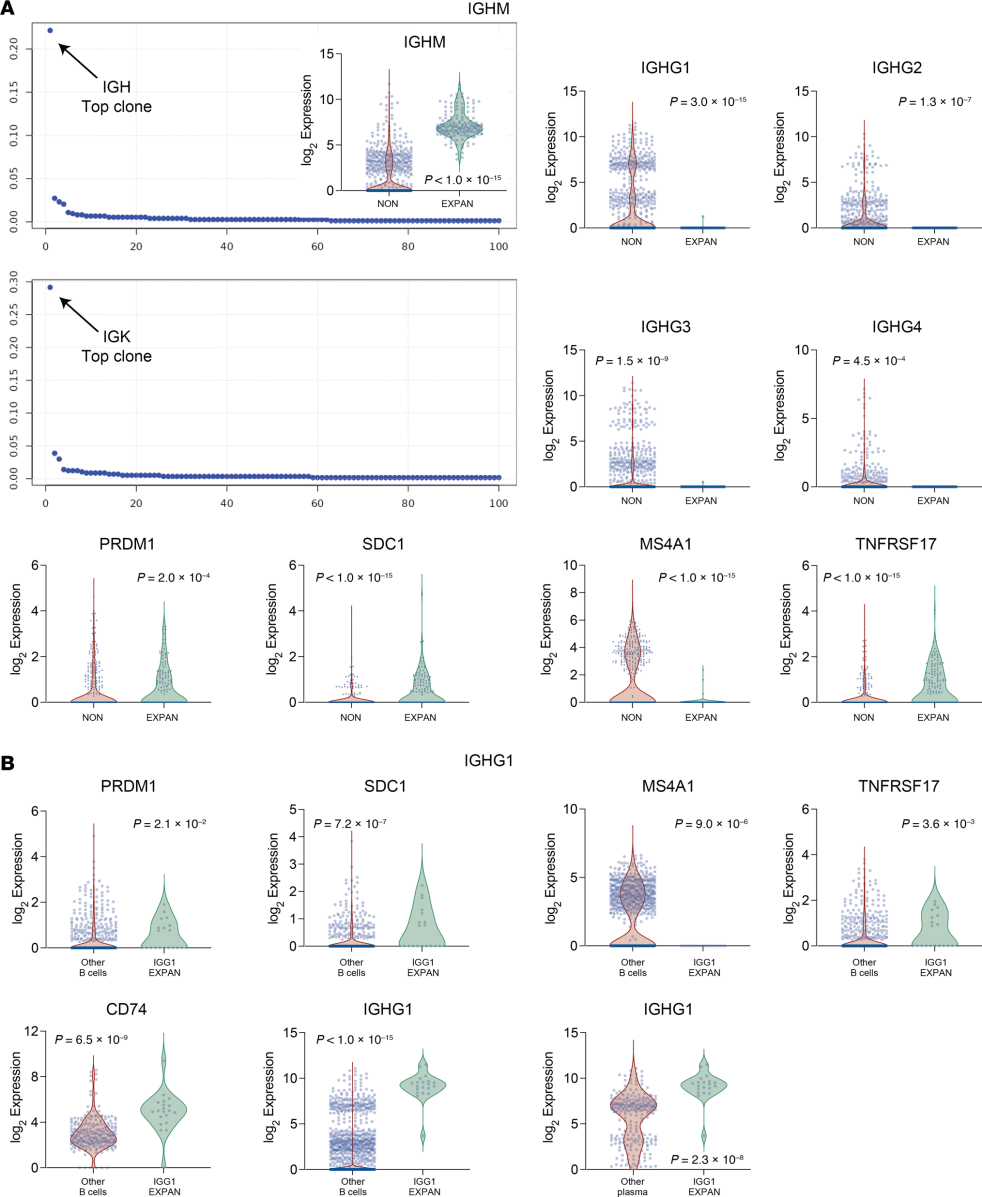
Single-cell B cell receptor gene sequencing (scBCR-seq) identifies clonal expansions of IGHM- and IGHG1-expressing plasma cells. (**A**) Rank order plots showing the frequency of individual B cell expansions in NL lesional skin. Arrows highlight the most abundant expanded clone based on IGH and IGK hypervariable gene segment frequencies. Violin plots depict the expression of genes relevant to B cell differentiation. The analysis reveals that the top-ranked clone is an IGHM-expressing plasma cell. (**B**) Gene expression analysis of the second most abundant B cell clonal expansion, an IGHG1-expressing plasma cell.

Under appropriate conditions, when T cells or B cells encounter their cognate antigen, they become activated and undergo repeated rounds of proliferation, resulting in clonal expansions of the initially activated immune cell. Thus, to determine whether the T and B cell infiltrate in NL lesional skin was due to purely increased migration, or if there were actual T and B cell expansions present, we performed single-cell BCR (scBCR) and scTCR sequencing. This technique identified various clonal B and T cell expansions in NL lesional skin (Figure 8A and Supplemental Figure 14). Because scRNA-seq was performed on the same sample set, we were then able to integrate the datasets and identify the gene expression profile of the expanded B and T cell populations. This analysis revealed that clonal expansions of *CCL5*-expressing NKG7 T cells were present in NL lesional skin (Supplemental Figure 14). It also demonstrated that an *IGHM*-expressing plasma cell was the strongest B cell expansion in NL lesional skin (Figure 8A). scBCR-seq also identified *IGHG1*-expressing clonal B cell expansions, which were also plasma cells based on their marker gene expression (Figure 8B).

### SPP1 is the dominant signaling pathway in NL.

**Figure 9:**
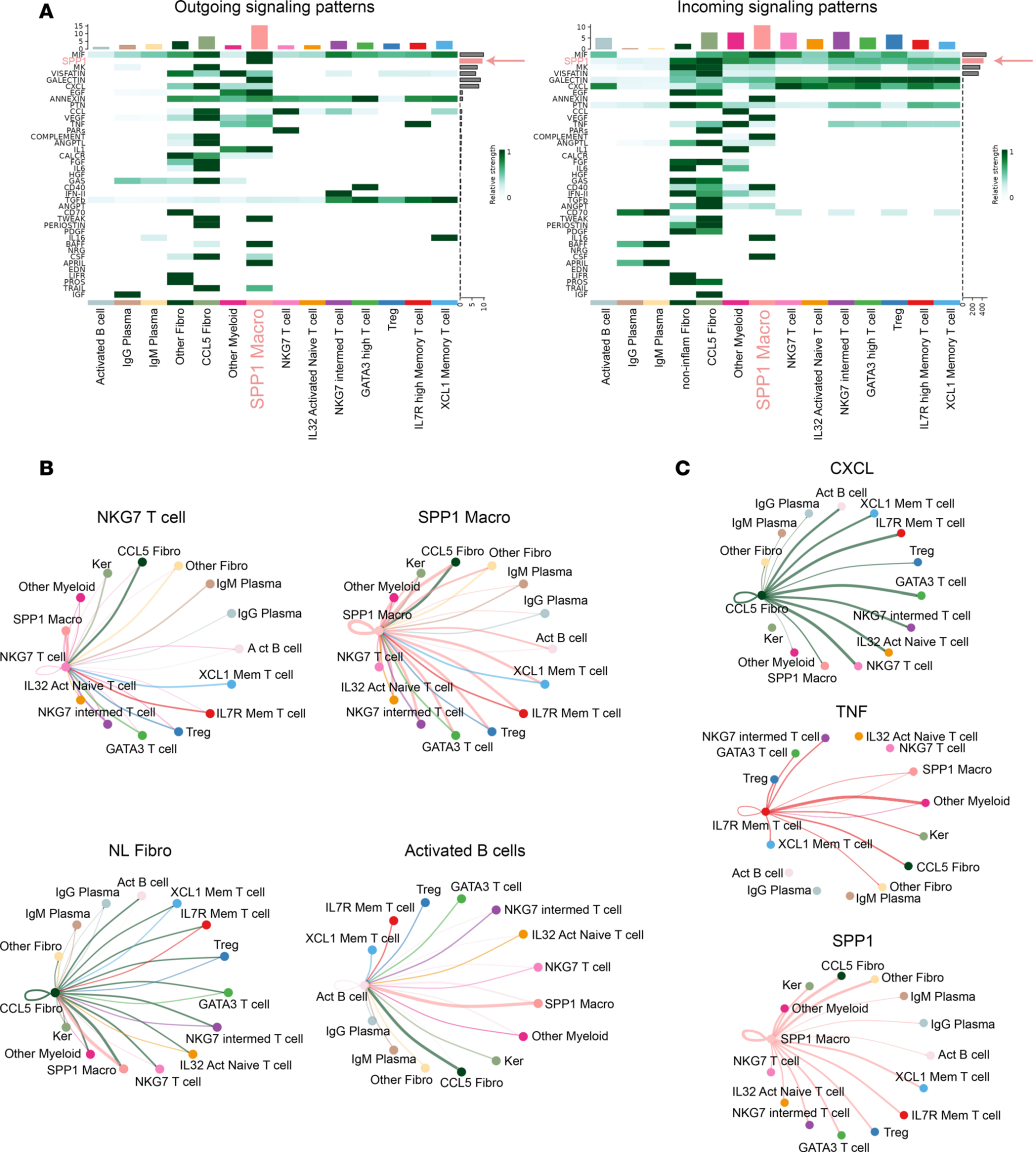
SPP1 is the dominant signaling pathway in NL. (**A**) To quantify cell-cell communications across aggregated cell groups, CellChat (9) was used to integrate gene expression analysis of signaling ligands, receptors, and their cofactors. The contributions of the different outgoing (left) and incoming (right) signals are presented as a heatmap, with signal intensities (rows) for each aggregated cell group (columns). Aggregate groups correspond to the following UMAP cell clusters: CCL5^hi^ fibroblasts (Fibroblast Clusters 6 and 8), Non-Inflammatory Fibroblasts (Fibroblast Clusters 1, 2, 3, 4, 5, and 7), Activated B cells (B cell Clusters 1 and 2), IgG Plasma cells (B cell Cluster 3), IgM Plasma cells (B cell Cluster 4), SPP1^hi^ macrophages (Myeloid Cluster 2), Other Myeloid (Myeloid Clusters 1, 3, and 4), NKG7 T cells (T cell Cluster 2), IL-32–activated Naive T cells (T cell Cluster 1), GATA3^hi^ T cells (T cell Cluster 4), T regulatory cells (T cell Cluster 6), IL-7R^hi^ Memory T cells (T cell Cluster 7), and XCL1^hi^ Memory T cells (T cell Cluster 8). SPP1^hi^ macrophages (SSP1 Macro) were the major aggregate group contributing to outgoing signals (orange bar), with the SPP1 pathway being the most dominant contributor to this outgoing signal (orange arrow). (**B**) Cell-cell communications between different cell aggregates are presented as circle plots. The thickness of the connecting lines represents the strength of each communication. (**C**) Signal pathway–specific analysis for CXCL chemokines, TNF, and SPP1. The thickness of the connecting lines represents the strength of each communication, with data presented as circle plots.

To quantify the biological significance of cell-cell communications across aggregated cell groups, we used CellChat to calculate the probability of cell-cell communication by integrating gene expression of signaling ligands, receptors, and their cofactors (9). To evaluate the signals contributing to outgoing communications of the different aggregated cell groups, a heatmap of signal intensities (rows) for each aggregated cell group (columns) was constructed. This analysis identified SPP1^hi^ macrophages as the major contributor of outgoing signals (Figure 9A, left side, peach bar). Of the signaling pathways that were differentially expressed in NL lesional skin, the SPP1 pathway contributed the most to this outgoing signal (Figure 9A, left side, peach arrow). A similar analysis was then performed for incoming communications (Figure 9A, right side). This second heatmap demonstrates that, with the exception of plasma cells, most cell aggregates received strong incoming signals across multiple different signaling pathways. For example, each NL-specific cell aggregate (CCL5^hi^ fibroblasts, SPP1^hi^ macrophages, and NKG7 T cells) received incoming CXCL and SPP1 pathway signals. Taking all cell aggregates into consideration, among NL differentially expressed pathways, the SPP1 pathway had the strongest average contribution to incoming signals (Figure 9A, right side, peach arrow).

Cell-cell communications between different cell aggregates were also quantified, and the results are displayed as circle plots, where the thickness of the connecting lines represents the strength of each communication (Figure 9B). This analysis revealed that most aggregate cell populations sent signals to NKG7 T cells, with the strong incoming signals coming from CCL5^hi^ fibroblasts and SPP1^hi^ macrophages. And, with the exception of plasma cells, NKG7 T cells sent outgoing signals to all cell types, with the strongest outgoing signal directed to SPP1^hi^ macrophages. Similarly, SPP1^hi^ macrophages also received strong incoming signals from most of the aggregated cell groups, except for the B cell subsets. The strongest incoming signal to SPP1^hi^ macrophages was from CCL5^hi^ fibroblasts (Figure 9B). SPP1^hi^ macrophages also sent very strong outgoing signals to all aggregate cell groups, especially T cell subsets and CCL5^hi^ fibroblasts. When cell-cell communications were analyzed separately for CCL5^hi^ fibroblasts, it became clear that these cells received strong incoming and outgoing communications with all other cell aggregates, except plasma cells. The strongest reciprocal communications were between CCL5^hi^ fibroblasts and SPP1^hi^ macrophages, highlighting the importance of these 2 cell populations in the pathophysiology of NL.

Additional analysis was performed to quantify the strength of specific signaling pathways (CXCL chemokines, TNF, and SPP1) between cell aggregate groups. CCL5^hi^ fibroblasts were high expressors of *CXCL9* (Supplemental Figure 6A). A circle plot of CXCL signaling from CCL5^hi^ fibroblasts demonstrates that there were strong CXCL communications between CCL5^hi^ fibroblasts and most other cell groups, except plasma cells and keratinocytes. In particular, there was very strong outgoing CXCL signaling from CCL5^hi^ fibroblasts to the T cell and activated B cell subsets.

With regard to other signaling pathways, IL-7R^hi^ memory T cells highly expressed *TNF* (Figures 6C and 9C). There was particularly strong *TNF* outgoing signaling from IL-7R^hi^ memory T cells to fibroblast, keratinocyte, and macrophage cell aggregates, with non–SPP1-expressing macrophages being the strongest recipients (Figure 9C, circle plot). Finally, as could be predicted from Figure 9A, SPP1 signaling from SPP1^hi^ macrophages was directed to all cell aggregates, with fibroblast subsets receiving the strongest SPP1 signal (Figure 9C, circle plot).

## Discussion

Our study revealed several characteristic features of necrobiosis, with 2 particularly striking findings: the expansion of specific immune cell populations in NL lesional skin and the elevated expression of various collagen genes that are typically poorly expressed in healthy skin. Among the expanded cell populations, the following stood out due to their rarity in healthy skin: NKG7 T cells, which express *CCL5*, *CD8A*, and cytotoxic effector genes; SPP1^hi^ macrophages, which express *CCL5* and *SPP1*; IGHM-expressing plasma cells; and CCL5^hi^ myofibroblasts, which express a range of collagens, collagenases, collagenase inhibitors, and inflammatory cytokines. Below, we discuss each of these findings in detail.

Deconvolution analysis of bulk RNA-seq and scRNA-seq highlighted a strong expansion of the T cell compartment in NL lesional skin (Figure 3B and Supplemental Figure 1). Although most T cell subsets were increased in NL, NKG7 T cells were of particular interest because they expressed several immune genes that were notably upregulated in NL, such as *CCL5* (Figure 6E). NKG7 T cells were also found to be clonally expanded in NL lesional skin (Supplemental Figure 14), but only represented approximately 3% of the T cell repertoire in healthy skin and were not increased in systemic sclerosis lesional skin (Figure 6G). They were found to be expanded in sarcoidosis, but Th1 cells expressing high levels of *STAT1* and *IFNG* predominated in that disease (Supplemental Figure 12).

NKG7 plays a critical role in the effector functions of cytotoxic T cells, particularly in the exocytosis of cytotoxic granules (10). Although not well characterized in healthy skin or other dermatologic conditions, NKG7 T cells have been studied in other settings, including anticancer and antiviral immune responses. Cancer patients with low NKG7 expression tend to be poor responders to immunotherapy, whereas immunotherapy responders often exhibit expansions of NKG7-expressing T cells (11, 12). In our study, NKG7 T cells detected in NL and NXG lesional skin were high expressors of the granzyme genes and *PRF1* (Figure 6E), both encoding cytotoxic effector molecules. While the specific cytotoxic target of NKG7 T cells in necrobiosis remains undetermined, scRNA-seq revealed that these cells are likely contributing to NL and NXG pathophysiology via their secretion of inflammatory cytokines, notably, *CCL5*, *IFNG*, and *IL32* (Figure 6E and Supplemental Figure 11). IHC analysis confirmed strong upregulation of CCL4 and CCL5 proteins in NL lesional lymphocytes (Figure 2B and Supplemental Figure 2).

CCL4 and CCL5, expressed by NKG7 T cells, are chemoattractants that recruit T cells, monocytes, macrophages, neutrophils, and DCs — all of which were expanded in NL lesional skin (Figure 2, A and B, and Supplemental Figure 1). In addition to its chemoattractant properties, CCL5 induces fibroblasts to express α-SMA (*ACTA2*) (13), which might explain the elevated *ACTA2* expression detected in NL fibroblasts (Supplemental Figure 4). NKG7 T cells also express *IFNG* and *IL32*, both encoding cytokines that promote monocyte to macrophage differentiation (14), and IFN-γ also induces macrophages to express SPP1 (15). A strong link between NKG7 T cells and SPP1-expressing macrophages in NL was further supported by cell-cell ligand-receptor analysis, which demonstrated reciprocal signaling between these 2 populations (Figure 9B, far left). Thus, the cytokines expressed by NKG7 T cells align well with the immunologic features of NL and NXG, including the formation of inflammatory SPP1-expressing macrophages.

scRNA-seq and FRP analysis also identified several other T cell populations that were expanded in NL and NXG lesional skin. Notably, there was a large population of recently activated *SELL*-, *IL32*-, and *CCR7*-expressing CD4^+^ T cells. Since skin-resident memory T cells do not express CCR7, the noted increase in *SELL*-, *IL32*-, and *CCR7*-expressing CD4^+^ T cells was likely due to recent activation of naive T cells. More mature Th1 cells expressing *IL2*, *TNF*, and *IFNG* were also common in NL lesional skin (Figure 6C, Cluster 5). These findings suggest that multiple T cell subpopulations may also contribute to the pathophysiology of NL and NXG.

M1 cytokine genes, such as *IL1A*, *IL1B*, and *IL6*, were modestly upregulated in NL macrophages, while the M2 cytokine gene *IL10* was slightly downregulated (Supplemental Figure 8B). However, the expression of genes encoding M1 and M2 cell surface markers was more variable. Therefore, characterizing NL as an M1-mediated disease may be an oversimplification. The most remarkable feature of NL and NXG macrophages was their high expression of *SPP1*. Thus, we designated this population as “SPP1^hi^ macrophages” to more accurately reflect their unique characteristics. Although SPP1^hi^ macrophages were a dominant feature in NL lesional skin, they were nearly absent in healthy and nonlesional skin, where they comprised only approximately 0.7% and approximately 1.4% of macrophages, respectively. These findings were confirmed at the protein level by OSTP IHC, which showed prominent OSTP staining in NXG and NL lesional skin macrophages (Supplemental Figure 3). SPP1^hi^ macrophages were not a predominant cell type in systemic sclerosis lesional skin (Figure 5H), but were present in sarcoidosis (Supplemental Figure 10).

Although not well characterized in skin, OSTP (encoded for by *SPP1*) has been described as an effector molecule in other settings, with notable roles in fibrosis. For instance, OSTP can induce fibroblasts to upregulate α-SMA and COL1A1, key markers of fibrosis (16). Recent studies have further highlighted the role of SPP1-expressing macrophages in driving myofibroblast activation, particularly in murine models of fibrosis (17). Earlier work also implicated *SPP1* as a driver of fibrosis, in particular a study linking bleomycin-induced pulmonary fibrosis to *SPP1*-expressing alveolar macrophages (18). Based on these findings, we hypothesize that the expansion of SPP1^hi^ macrophages in NL and NXG lesional skin plays a critical role in driving NL pathophysiology. This hypothesis is supported by cell-cell ligand-receptor analysis, which revealed strong reciprocal signaling between SPP1^hi^ macrophages and CCL5^hi^ fibroblasts (Figure 9B). Additionally, in terms of outgoing signals, SPP1 emerged as the most strongly upregulated signaling pathway in lesional compared with nonlesional NL skin (Figure 9A). Taken together, across multiple independent sample sets and experimental approaches, our results show that *SPP1* appears to be a key effector pathway in NL and NXG.

In addition to *SPP1*, NL macrophages also expressed other genes encoding effector cytokines, including *CCL5*, *CXCL9*, and *CXCL10*. Like CCL5, CXCL9 has been linked to increased collagen production and fibrosis (19–21). SPP1^hi^ macrophages also express ECM genes, such as *FN1* (Figure 5F), which has been studied as a biomarker of NL (22). Furthermore, SPP1^hi^ macrophages showed marked upregulation of several genes involved in immune and complement responses, including *C1QA*, *C1QC*, *FCGR1A*, *FCGR3A*, and *MRC1* (Supplemental Figure 8A). *C1QA* and *C1QC* both encode complement proteins that bind to the Fc region of immunoglobulins. Given that both IgM and C3 are known to be deposited in NL lesional skin (23), it is likely that C1 produced by SPP1^hi^ macrophages contributes to this deposition.

Our results provide insights into the nature of the B cell infiltrate associated with necrobiosis. Bulk RNA-seq revealed upregulation of *IGHM* and *JCHAIN* in NL and NXG lesional skin samples compared with paired nonlesional controls (Figure 1A). Furthermore, many of the *IGHM*-expressing cells in NL lesional skin were plasma cells (Figure 7C) and scBCR-seq revealed strong clonal expansions within this population (Figure 8A). This is a surprising discovery because, under typical circumstances, activated B cells differentiate into plasma cells after they have undergone isotype switching, a process in which the BCR’s constant region is exchanged from IGHM to either IGHA, IGHG, or IGHE. Therefore, plasma cells do not usually retain IgM expression. Additionally, human skin generally contains very few B cells, yet in NL, B cells represent a substantial component of the lesional infiltrate, comprising approximately 10% of all cells in NL lesional skin. Although this percentage may seem large, B cells are increasingly recognized as playing roles in various immune-mediated skin diseases, such as hidradenitis suppurativa, systemic sclerosis, and erythema migrans (24, 25).

To emphasize the uniqueness of our findings, we confirmed that *IGHM*-expressing plasma cells were absent in systemic sclerosis and sarcoidosis (Supplemental Figure 13, A and D). While *IGHM*-expressing plasma cells have not been well characterized in human skin, they have been reported in mice (26). We hypothesize that necrobiotic skin provides a unique microenvironment that enables plasma cell differentiation without the typical isotype switching. Specifically, the cytokines that promote isotype switching (e.g., IL-4, IL-13, and IFN-γ) are only poorly to modestly expressed in necrobiotic skin when compared with other inflammatory conditions like atopic dermatitis. This may explain why B cells can mature without undergoing isotype switching in NL. Additionally, given that IgM and complement deposition are hallmarks of necrobiotic skin, *IGHM*-expressing plasma cells likely contribute to the pathophysiology of necrobiosis.

Although traditionally thought of as purely stromal cells, the role of fibroblasts in immunological processes has become increasingly apparent, particularly in immune-mediated skin diseases (25, 27–30). Of the various cell types associated with necrobiosis, the most intriguing were the CCL5^hi^ fibroblasts (Figure 4A; Cluster 8, and to a lesser extent, Cluster 6), which were exclusively derived from NL lesional skin. These CCL5^hi^ fibroblasts also expressed other inflammatory cytokines, including *CXCL9*, *IL32*, and *MDK* (Figure 4C). Like CCL5 and CXCL9, MDK is a profibrotic cytokine, demonstrated by its role in murine models of pulmonary fibrosis and its use as a biomarker in idiopathic pulmonary fibrosis (31). Unlike in NL and NXG skin, CCL5^hi^ fibroblasts are not a dominant feature of other inflammatory skin conditions. When compared with NL and NXG fibroblasts (Supplemental Figure 5), systemic sclerosis lesional fibroblasts showed a more modest upregulation of *CCL5* and *CXCL9* (Supplemental Figure 7A). While sarcoidosis fibroblasts also upregulated *CXCL9* and *CD74*, they exhibited only modest increases in *CCL5* and *FN1* (Supplemental Figure 7B). This contrasts with the strong correlation between *CCL5* and *CXCL9* in NL fibroblasts (Supplemental Figure 6A). However, the dissociation of *CXCL9* and *CCL5* expression in sarcoidosis fibroblasts is not surprising because different external cytokine stimuli are required to induce *CXCL9* and *CCL5* expression in fibroblasts (Supplemental Figure 6B).

It is traditionally thought that necrobiosis results from the “degeneration” of collagen fibers, but our data suggest that this view does not fully capture the molecular pathophysiology of the disease. Although *MMP9*, a collagenase-encoding gene, was upregulated in NL, its inhibitor, *TIMP1*, was also highly expressed. This finding indicates that NL is not simply a disease of unopposed collagen degradation. While collagen degradation likely plays a role in the necrobiosis phenotype, a more striking observation was that CCL5^hi^ fibroblasts upregulated expression of ECM genes that are typically poorly expressed in healthy skin. Based on these findings, we propose that necrobiosis is, in large part, driven by changes in ECM composition. Specifically, bulk RNA-seq, scRNA-seq, and FRP consistently revealed strong upregulation of additional collagen genes (e.g., *COL4A4*, *COL6A3*, *COL8A1*, and *COL11A1*) in NL lesional skin, specifically in CCL5^hi^ fibroblasts. These fibroblasts also showed marked upregulation of other ECM genes (e.g., *POSTN*, *FN1*, and *TNC*) known to be linked to fibrosis. For example, POSTN is highly expressed in fibroblasts from patients with pulmonary fibrosis, and POSTN-deficient mice exhibit resistance to bleomycin-induced pulmonary fibrosis (32). Fibronectin has several isoforms. Its EDA isoform has been implicated in fibrosis through its ability to bind integrins and Toll-like receptor 4 (TLR4) (33). Like fibronectin, TNC also binds TLR4 and has been associated with various fibrotic diseases across multiple organs (34). Its profibrotic properties are partly mediated by its ability to stimulate fibroblasts to upregulate collagen expression and differentiate into myofibroblasts (35).

**Figure 10:**
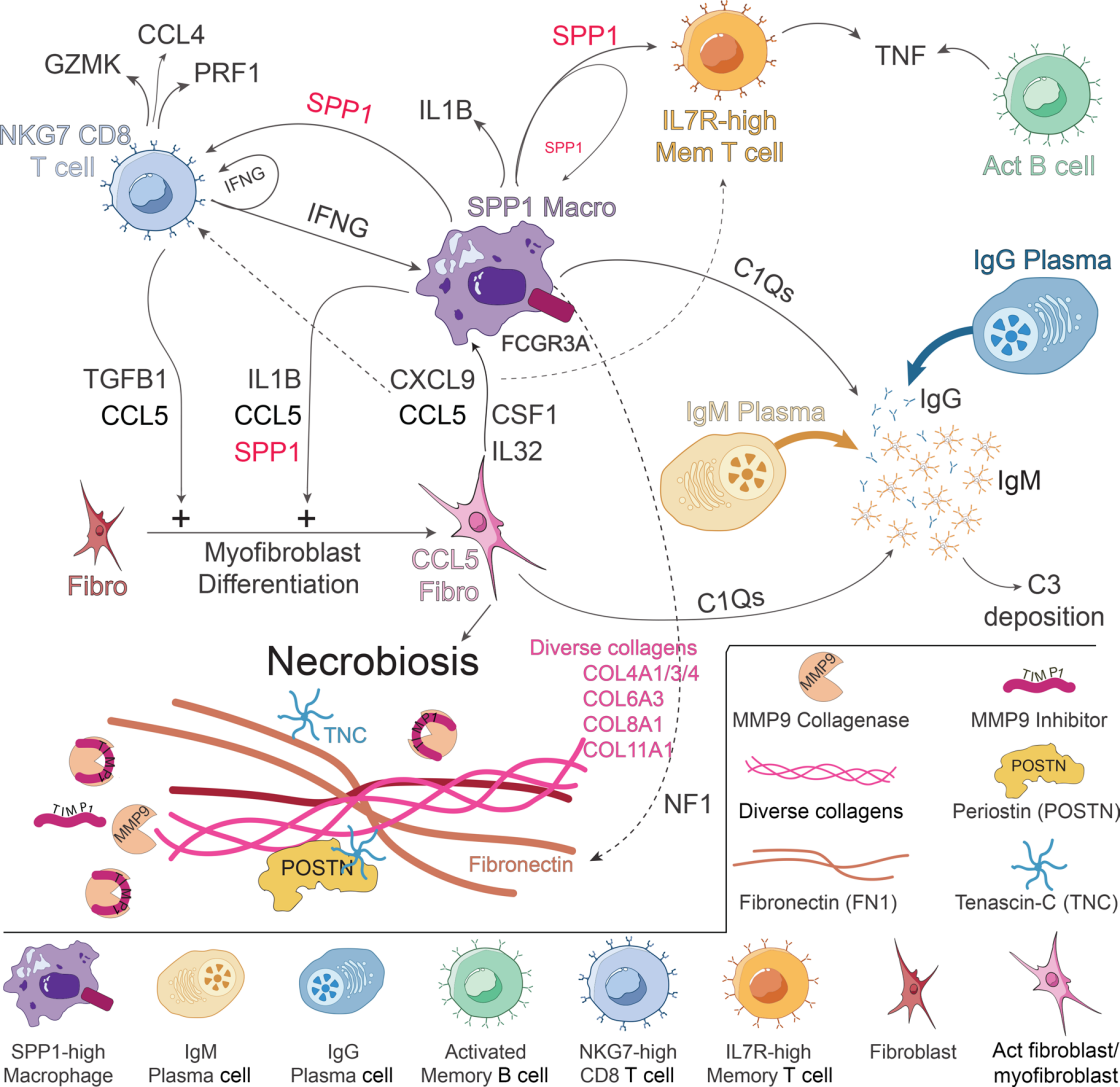
Graphical representation of necrobiosis pathophysiology. SPP1^hi^ macrophages are key mediators of NL, producing profibrotic and proinflammatory mediators such as SPP1 and IL-1β. SPP1 supports T cell survival and promotes IFN-γ production by T cells. Additionally, SPP1 has autocrine functions, enhancing macrophage production of inflammatory mediators, including IL-1β. SPP1 also induces fibroblast differentiation into proinflammatory fibroblasts and myofibroblasts. Additionally, SPP1^hi^ macrophages express complement proteins (C1Qs). CCL5^hi^ fibroblasts are profibrotic cells that communicate with SPP1^hi^ macrophages through the production of CXCL9, CCL5, CSF1, and IL-32. CXCL9 and CCL5 also act as chemoattractants for T cells. CCL5^hi^ fibroblasts upregulate the production of various extracellular matrix proteins, including diverse collagens, POSTN, FN1, TNC, MMP9, and the MMP9 inhibitor TIMP1. Like SPP1^hi^ macrophages, CCL5^hi^ fibroblasts also strongly express C1Qs. IgM and IgG plasma cells produce their respective immunoglobulins, and in conjunction with C1Q, contribute to C3 deposition. NKG7 T cells communicate with SPP1^hi^ macrophages through IFN-γ production and help differentiate fibroblasts into CCL5^hi^ fibroblasts through the secretion of CCL5 and TGF-β1. Additional cytokines, including IFN-γ, TGF-β1, and TNF, are produced by various T cell subsets, such as the TNF-producing IL-7^hi^ memory T cells. Activated memory B cells also produce inflammatory cytokines, including TNF.

Finally, in addition to being a major source of profibrotic cytokines and ECM proteins, NL-specific CCL5^hi^ fibroblasts (Figure 4A, Cluster 8) also strongly upregulated MHC class II genes and the MHC-associated gene *CD74* (Figure 4C). This suggests that this fibroblast subpopulation may also interact directly with T cells, a hypothesis supported by cell-cell ligand-receptor analysis, which demonstrated strong connections between CCL5^hi^ fibroblasts and multiple T cell clusters (Figure 9B). Figure 10 summarizes the pathways involved in necrobiosis based on the key findings of our investigation.

To date, no highly effective therapies have been identified for NL. Both JAK inhibitors and TNF blockade have been used, with limited success (36, 37). Our results show that in NL and NXG SPP1 is the predominant signaling and CCL5 is highly expressed by 3 distinct immune cell subtypes in lesional skin — NKG7 T cells, SPP1^hi^ macrophages, and CCL5^hi^ fibroblasts. Based on these findings, we propose SPP1 and CCL5 as therapeutic targets for NL and NXG.

## Methods

### Sex as a biological variable.

Both male and female study participants were randomly selected. Sex was not considered a biological variable in the analysis.

### Samples.

Fresh specimens were obtained from patients seen at the University of California, Davis, Dermatology Clinic. For bulk RNA-seq, 4-mm punch biopsies were collected from lesional (NL, *n* = 3; NXG, *n* = 3), nonlesional (NL, *n* = 3; NXG, *n* = 3), and healthy control (*n* = 3) skin. For fresh scRNA-seq, scBCR-seq, and scTCR-seq, samples were obtained from NL lesional (*n* = 1), NL nonlesional (*n* = 1), and healthy (*n* = 1) skin. Archived formalin-fixed paraffin-embedded (FFPE) specimens were obtained from the University of Michigan. For single-cell gene expression FRP, samples were collected from lesional skin (NL, *n* = 8; NXG, *n* = 8) and healthy control skin (*n* = 4). IHC for CCL4, CCL5, CXCL9, and IL-32 was performed on samples from NL lesional skin (*n* = 12) and healthy control skin (*n* = 3). For SPP1 IHC, samples were obtained from lesional skin (NL, *n* = 6; NXG, *n* = 6) and healthy control skin (*n* = 3). Systemic sclerosis skin samples (*n* = 4) and control healthy skin samples (*n* = 3) for scRNA-seq were obtained from the University of Michigan.

### IHC staining.

FFPE skin blocks were sectioned into 5-µm slices. The sections were incubated at 60°C for 30 minutes to remove excess paraffin. Slides were placed in PH9 antigen retrieval buffer and heated at 125°C for 30 seconds in a pressure cooker. Individual sections were stained with anti-CCL4 (Thermo Fisher Scientific, PA5-23681), anti-CCL5 (R&D Systems, AF-278-SP), anti-CXCL9 (R&D Systems, AF392), anti–IL-32 (MiiliporeSigma, HPA029397), or anti-OSTP (Thermo Fisher Scientific, MA5-17180) antibodies. Antibody incubation was performed for overnight at 4°C, using a Diaminobenzidine Substrate Detection system (Thermo Fisher Scientific), followed by counterstaining with hematoxylin.

### RNA-seq.

Total RNA was extracted using the RNeasy Plus Mini Kit (Qiagen, 74134). RNA concentrations were quantified using a Qubit Fluorometer (Invitrogen), and RNA integrity was assessed using the Agilent TapeStation. Only samples with an RNA integrity number (RIN) of 8 or higher were used for this study. Indexed libraries were constructed from 500 ng of total RNA using the TruSeq Stranded mRNA Sample Prep Kit (Illumina, 20020594) following the manufacturer’s instructions. Library quantity and quality were assessed by Qubit and the Agilent 2100 Bioanalyzer, respectively. The average library size was 400 bp. Libraries’ molar concentrations were validated by qPCR prior to library pooling. Sequencing was performed on the Illumina NovaSeq 6000 platform using PE150 chemistry (Illumina).

### scRNA-seq.

Single-cell suspensions were prepared, and scRNA-seq libraries were generated using the Chromium Next GEM Single Cell 5′ HT v2 Kit. During library preparation, samples were split after cDNA synthesis, and scTCR and scBCR libraries were prepared using the Chromium Single Cell Human TCR/BCR Amplification Kit. scRNA-seq libraries, along with the scTCR and scBCR libraries, were sequenced on the Illumina NovaSeq 6000 sequencer and the NovaSeq X Plus NovaSeq X Plus 25B, respectively, to generate 151-bp paired-end reads. Data processing, including quality control, read alignment, and gene quantification, was performed using 10× Cell Ranger software (v7.1.0). The Seurat R package (v5.0.1) (https://cran.r-project.org/web/packages/Seurat/index.html) was used for normalization, data integration, clustering analysis, differential gene expression analysis, and UMAP.

### Statistics.

For scRNA-seq, the Seurat R package (v5.0.1) was used for normalization, data integration, clustering analysis, differential gene expression analysis, and UMAP. Cell types were identified using SingleR (v2.2.0) (38). Additionally, clustered cells were verified by matching cell cluster gene signatures with known cell-type-specific markers. CellChat (v1.6.1) was used to infer cell-cell communication (9).

For bulk RNA-seq, gene expression normalization and differential expression analysis were performed using the DESeq2 Bioconductor R package (v1.40.2) (https://bioconductor.org/packages/release/bioc/html/DESeq2.html). This package employs a model based on the negative binomial distribution to calculate differential expression statistics. Differential expression *P* values were corrected for multiple testing using the false discovery rate (FDR) method. Genes with adjusted *P* values of less than 0.05 and FC greater than 2 or less than 0.5 were considered differentially expressed. PCA was performed on regularized log-transformed data created with DESeq2. The original count data were transformed to the log_2_ scale to minimize differences between samples for rows with small counts while normalizing for library size. After transformation, the top 500 rows with the highest variance were selected for PCA. Clustered heatmaps of the regularized log-transformed data were created using the “pheatmap” R package. For further clustering and heatmap analysis, the top 30 most highly variable genes were selected.

### Study approval.

The study was approved by the UC Davis Institutional Review Board (IRB). All participants provided written informed consent prior to participation.

### Data availability.

The NL bulk RNA-seq and scRNA-seq datasets, systemic sclerosis scRNA-seq dataset, and the NXG and NL single-cell FRP datasets described in this manuscript have been deposited in the NCBI Sequence Read Archive (SRA) under BioProject IDs PRJNA1181556, PRJNA1182033, and PRJNA1048434. scRNA-seq FASTQ files from sarcoidosis lesional skin were obtained from a previously published dataset (NCBI GEO GSE234901) (39). Raw data underlying the results of this study can be found in the Supporting Data Values file. All other data and materials generated as part of this work will be made available upon request.

## Author contributions

EM, JEG, AIM, AAM, JMK, LCT, and IEA conceptualized the study. EM, AIM, STL, and AAM developed the methodology. EM, STL, AIM, AAM, A Kirane, OK, A Kunitsyn, NYK, WL, SYL, XX, AG, LD, SM, ACB, PWH, and MCB carried out the investigation. EM provided project administration. EM, JEG, and LCT supervised the project. All authors wrote, reviewed, and edited the manuscript.

## Supplementary Material

Supplemental data

Supporting data values

## Version 1 02/24/2025

Electronic publication
