# Supplementary material for: SPP1hi macrophages, NKG7 T cells, CCL5hi fibroblasts, and IgM plasma cells are dominant features of necrobiosis
Source: JCI Insight. 2025 Feb 24;10(4):e178766. doi: 10.1172/jci.insight.178766 (PMC11949047; doi:10.1172/jci.insight.178766)
Supplement: Supplemental data [file jciinsight-10-178766-s141.pdf]

SUPPLEMENTAL FIGURE LEGENDS

Supplemental Figure 1. Deconvolution analysis of NL and NXG bulk RNA-seq data.

Supplemental Figure 2. Immunohistochemistry (IHC) analysis of NL lesional and control healthy skin. IHC analysis was performed on paraffin embedded tissue obtained from the University of Michigan. NL patients were compared to healthy skin controls. IHC results for CCL4, CCL5, IL32, and CXCL9 are shown.

Supplemental Figure 3. Immunohistochemistry (IHC) analysis of NL and NXG lesional skin reveals strong SPP1 expression. IHC of SPP1 (aka OSTP or Osteopontin) was performed on paraffin embedded tissue obtained from the University of Michigan.

Supplemental Figure 4. Cluster 8 fibroblasts have higher expression of myofibroblast markers ACTA2, CTGF, and PLOD2. Additional analysis of fibroblast Clusters 6 and 8 from Figure 3A are shown. Violin plot of gene expression for the corresponding scRNA-seq clusters are presented for the indicated genes. Each dot represents a single cell.

Supplemental Figure 5. Single-cell gene expression flex fixed RNA profiling (FRP) of NL and NXG fibroblasts; NL and NXG lesional fibroblasts compared to healthy skin control fibroblasts. FRP pseudobulk analysis is presented as box-and-whisker plots. Each dot represents a single sample. The upper and lower bars connected to each box indicate the boundaries of the normal distribution and the upper and lower box edges mark the first and third quartile boundaries within each distribution. The bold line within the box indicates the median value of the distribution. Below each box-and-whisker plot is a violin plot for the corresponding scRNA-seq data in which each dot represents a single cell. For this analysis, all fibroblast clusters were binned together. Significance was determined by the Student's t-test.

Supplemental Figure 6. Single-cell RNA-seq of lesional and non-lesional NL skin samples. A) Scatter plot of intracellular expression of CCL5 and CXCL9. Each dot represents a single fibroblast cell in NL lesional skin. B) Cultured fibroblast gene expression data. Primary fibroblasts were isolated from healthy skin and cultured with noted cytokines. Gene expression analysis was performed by bulk RNA-seq. *IL32* (upper plot), *CXCL9* (middle plot), and *CCL5* (lower plot) expression are shown as Log2 reads. C) The UMAP method was used to visualize scRNA-seq data of dermal cells isolated from systemic sclerosis and healthy skin. Each dot represents a single cell. Clusters containing fibroblast cells are colored. D) Violin plots representing each fibroblast cluster from "C" demonstrate that none of the fibroblast subclusters highly expressed *CCL5*. Other genes of interest are alos shown. Numbers in red represent clusters with a greater percentage of systemic sclerosis fibroblasts.

Supplemental Figure 7. scRNA-seq of systemic sclerosis and sarcoidosis fibroblasts. A) Systemic sclerosis scRNA-seq gene expression data for all fibroblast clusters binned together are presented. For each violin plot each dot represents a single cell. Systemic sclerosis fibroblasts are compared to healthy skin fibroblasts. B) *Sarcoidosis* scRNA-seq gene expression data for all fibroblast clusters binned together. Sarcoidosis fibroblasts are compared to healthy skin fibroblasts. Plots in which gene expression has the same directionality as in NL are shown. The upper box-and-whisker plots represent scRNA-seq pseudobulk analysis. Each dot represents a single sample. The upper and lower bars connected to each box indicate the boundaries of the normal distribution and the upper and lower box edges mark the first and third quartile boundaries within each distribution. The bold line within the box indicates the median value of the distribution. Below each box-and-whisker plot is a violin plot for the corresponding scRNA-seq data in which each dot represents a single cell. Significance was determined by the Student's T-test. C) Sarcoidosis scRNA-seq gene expression data as described in "B" except plots are for gene expression data with opposite directionality as NL or for comparisons that do not reach significance in the pseudobulk analysis.

Supplemental Figure 8. scRNA-seq of NL myeloid cells. A) Violin plots for indicated differentially expressed genes across seven myeloid clusters from UMAP shown in Figure 4. Each dot represents a single cell. B) NL macrophages from *all myeloid cell clusters in Figure 4 were binned together and compared to control macrophages* (healthy skin macrophages and non-lesional macrophages). Differentially expressed gene expression data is presented as violin plots where each dot represents a single cell.

Supplemental Figure 9. Single-cell gene expression flex fixed RNA profiling (FRP) reveals high expression of CCL5 and SPP1 in NL and NXG lesional macrophages compared to healthy skin control macrophages. FRP pseudobulk analysis is presented as box-and-whisker plots. Each dot represents a single sample. The upper and lower bars connected to each box indicate the boundaries of the normal distribution and the upper and lower box edges mark the first and third quartile boundaries within each distribution. The bold line within the box indicates the median value of the distribution. Below each box-and-whisker plot is a violin plot for the corresponding scRNA-seq data in which each dot represents a single cell. For this analysis all macrophage clusters were binned together. Significance was determined by the Student's T-test.

Supplemental Figure 10. scRNA-seq of sarcoidosis macrophages A) Sarcoidosis scRNA-seq - Plots in which gene expression has the same directionality as in NL are shown. scRNA-seq gene expression data for all sarcoidosis macrophage clusters were binned together and compared to healthy skin macrophages. scRNA-seq pseudobulk analysis is presented as box-and-whisker plots. Each dot represents a single sample. The upper and lower bars connected to each box indicate the boundaries of the normal distribution and the upper and lower box edges mark the first and third quartile boundaries within each distribution. The bold line within the box indicates the median value of the distribution. Below each box-and-whisker plot is a violin plot for the corresponding scRNA-seq data in which each dot represents a single cell. *B) Sarcoidosis scRNA-seq* expression data as described in "A" except plots are for gene expression data with opposite directionality as NL or for comparisons that do not reach significance in the pseudobulk analysis.

Supplemental Figure 11. Single-cell gene expression flex fixed RNA profiling (FRP) reveals increased CCR7, JAK3, SELL, and CCR7 in NL and NXG lesional T cells compared to healthy skin control T cells. A) The upper violin plots represent additional scRNA-seq gene expression data for NL lesional skin versus control (healthy skin and non-lesional skin). The samples analyzed were fresh biopsy specimens. All scRNA_seq T cell clusters were binned together for the analysis. Each dot represents a single cell. Below the scRNA-seq plots are results from the FRP analysis. FRP was performed on formalin fixed archived specimens. NL FRP pseudobulk results are presented as box-and-whisker plots. For this analysis all FRP T cell clusters were binned together. NL lesional skin is compared to healthy control skin. Each dot represents a single sample. The upper and lower bars connected to each box indicate the boundaries of the normal distribution and the upper and lower box edges mark the first and third quartile boundaries within each distribution. The bold line within the box indicates the median value of the distribution. Below each box-and-whisker plot is a violin plot for the corresponding scRNA-seq data in which each dot represents a single cell. Significance was determined by the Student's t-test. B) FRP gene expression data as described in "A" except for NXG samples. C) FRP gene expression analysis of NKG7-expressing NL lesional and healthy control skin T cells. This analysis differs from "A" and "B" in that only T cells expressing NKG7 were analyzed.

Supplemental Figure 12. ScRNA-seq gene expression profiling of sarcoidosis T cells highlights strong upregulation of STAT1 and IFNG. Upper violin plots- Additional scRNA-seq gene expression data for NL lesional skin versus control (healthy skin and non-lesional skin) is shown for comparison. All scRNA-seq T cell clusters were binned together for the analysis. Data is presented as violin plots where each dot represents a single cell. Below the NL scRNA-seq plots are the scRNA-seq plots for sarcoidosis T cells. Results of pseudobulk analysis are represented as box-and-whisker plots. For this analysis all sarcoidosis T cell clusters binned together. Each dot represents a single sample. The upper and lower bars connected to each box indicate the boundaries of the normal distribution and the upper and lower box edges mark the first and third quartile boundaries within each distribution. The bold line within the box indicates the median value of the distribution. Below each box-and-

whisker plot is a violin plot for the corresponding sarcoidosis scRNA-seq data in which each dot represents a single cell.

Supplemental Figure 13. A large percentage of IGHM-expressing B cells in NL lesional skin are plasma cells. A) Comparison of plasma cell markers expressed by *IGHM* B cells in NL and systemic sclerosis (Ssc) lesional skin. For this analysis *IGHM*-expressing B cells identified by scRNA-seq were analyzed. From within this population, plasma cells can be identified by their lack of *MS4A1* (encodes CD20) expression and the positive expression of one of the plasma cell markers- *PRDM1* (encodes BLIMP1), *SDC1* (encodes CD138), and *TNFRSF17* (encodes CD269). Venn diagrams are used for visual comparison of systemic sclerosis and NL *IGHM*-expressing B cells. Note that no *IGHM*-expressing B cells in systemic sclerosis (Ssc) are plasma cells, i.e. nearly all of the Ssc *IGHM*-expressing B cells express *MS4A1* and none express any of the plasma cell markers (*PRDM1, SDC1, or TNFRSF17*). B) Similar analysis to that described in "A" but for NL and NXG FRP datasets. Although the FRP technique is not as sensitive as scRNA-seq, FRP analysis was able to detect plasma cell markers in NL *IGHM*-expressing B cells. *IGHM*-expressing B cells in NXG have a similar phenotype to those in NL. C) scRNA-seq gene expression analysis of NL plasma cells demonstrates that most plasma cells in NL are either *IGHG1* or *IGHM* expressing. For this analysis all NL plasma cells were binned together and then *IGH* gene expression was assessed. In the violin plots each dot represents an individual plasma cell. D) Same scRNA-seq analysis described in "C" was performed for sarcoidosis. Note that practically no plasma cells were found in sarcoidosis lesional skin.

Supplemental Figure 14. Single-cell T cell receptor gene sequencing (scTCR) identifies clonal expansions of NKG7-expressing T cells. Top, Rank order plots illustrate the frequency of individual T cell expansions in NL lesional skin. Arrows indicate the most abundant expanded clones based on TCR alpha and beta hypervariable CDR3 sequence frequencies. Violin plots depict expression of genes relevant to NL pathophysiology or T cell differentiation. Note the top T cell expansion expresses low *ICOS*, *IL7R*, and *LTB* and high *IFNG*, *CCL5*, *NKG7*, *TNFRSF9*, and *LAG3*. Comparison of genes expressed by the most highly expanded T cells versus other NL lesional T cells is also presented as a volcano plot.

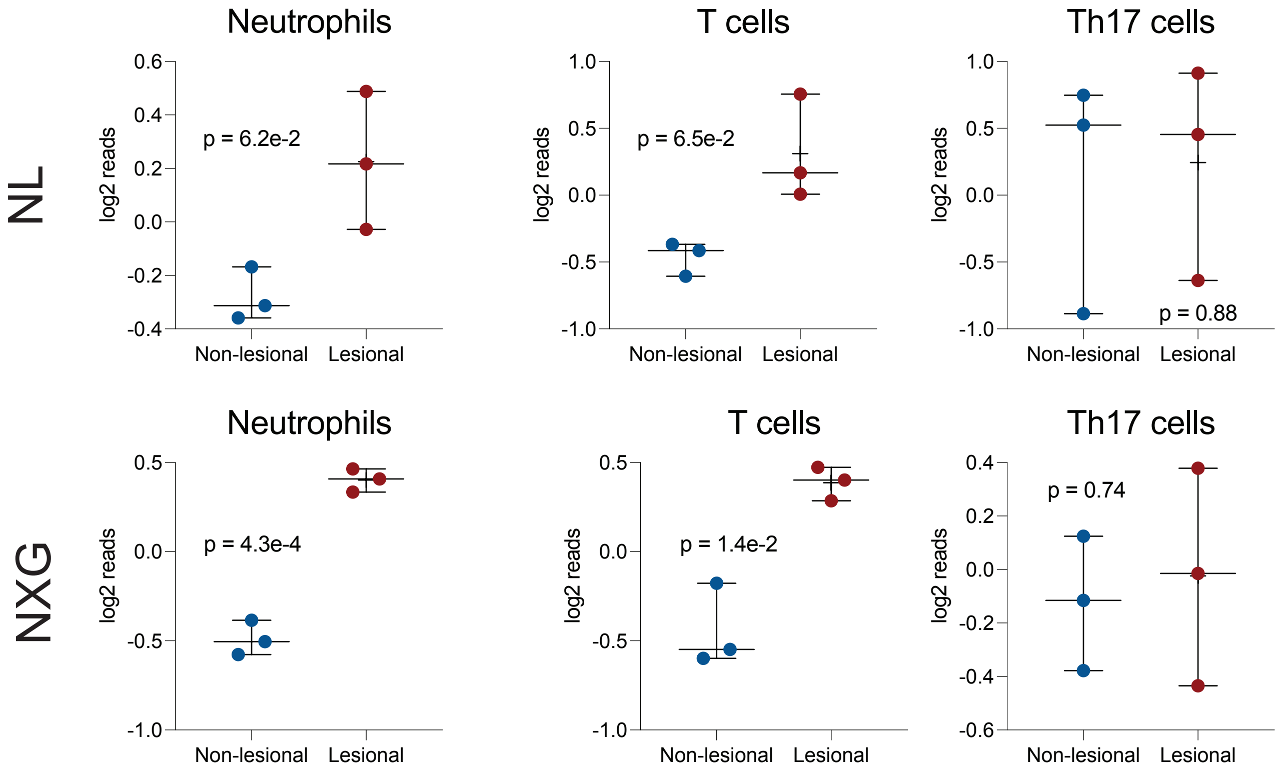
NL
NXG
Neutrophils
T cells
Th17 cells
log2 reads
p = 6.2e-2
p = 6.5e-2
p = 0.88
p = 4.3e-4
p = 1.4e-2
p = 0.74
Non-lesional
Lesional

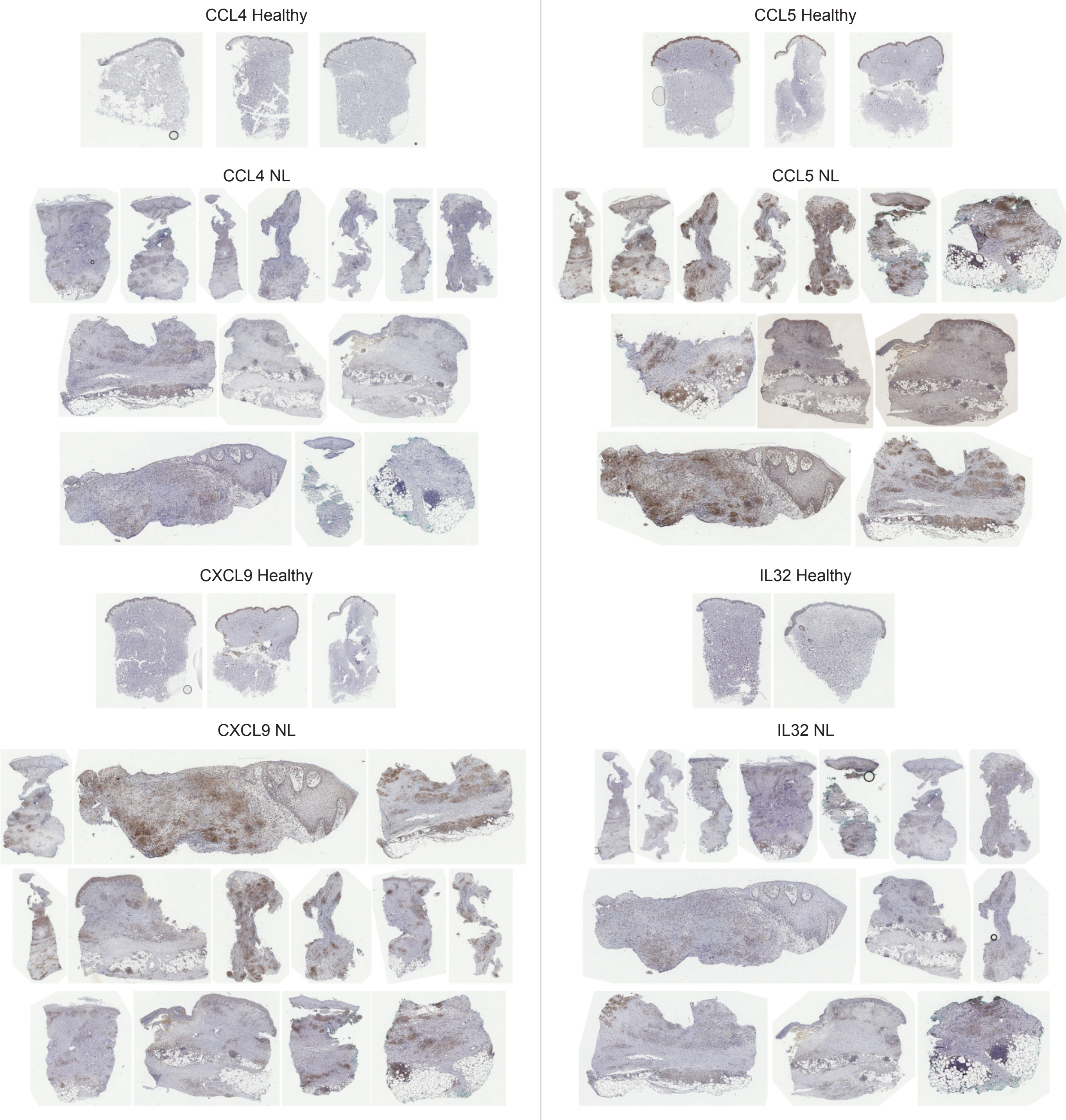

CCL4 Healthy
CCL4 NL
CCL5 Healthy
CCL5 NL
CXCL9 Healthy
CXCL9 NL
IL32 Healthy
IL32 NL

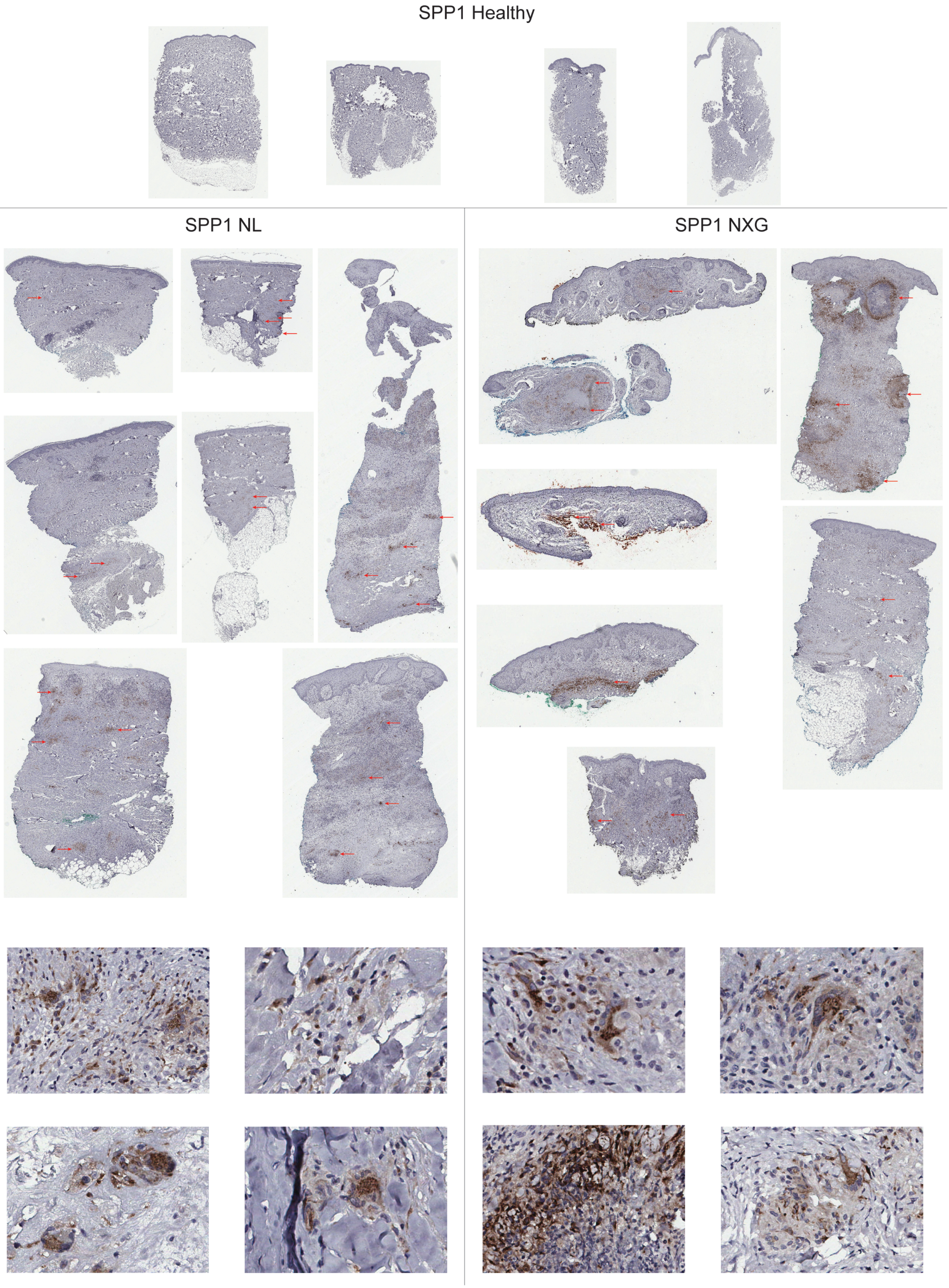
SPP1 Healthy
SPP1 NL
SPP1 NXG

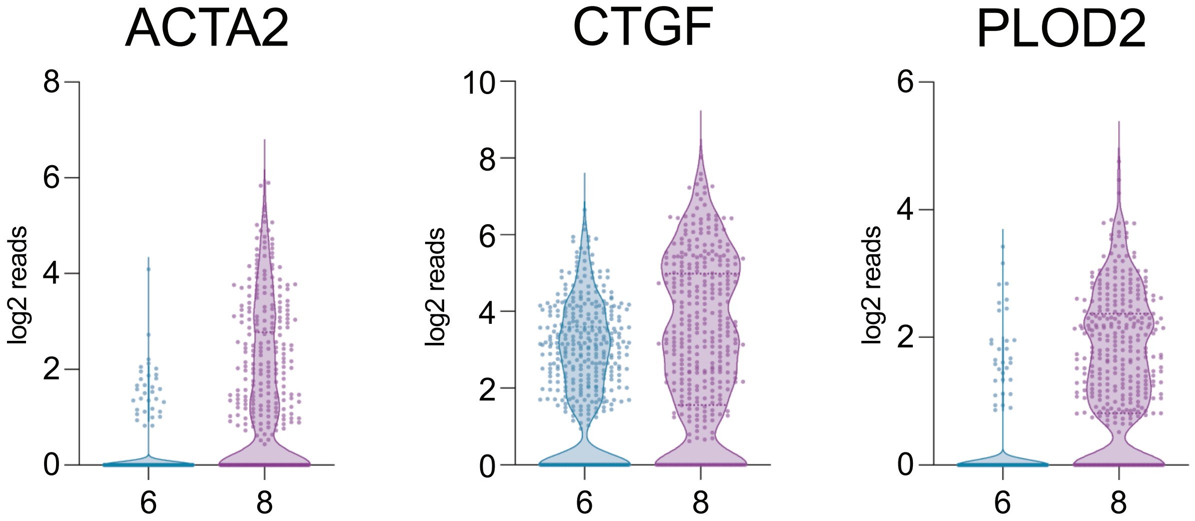
ACTA2
CTGF
PLOD2
log2 reads
8
6
4
2
0
10
6
8

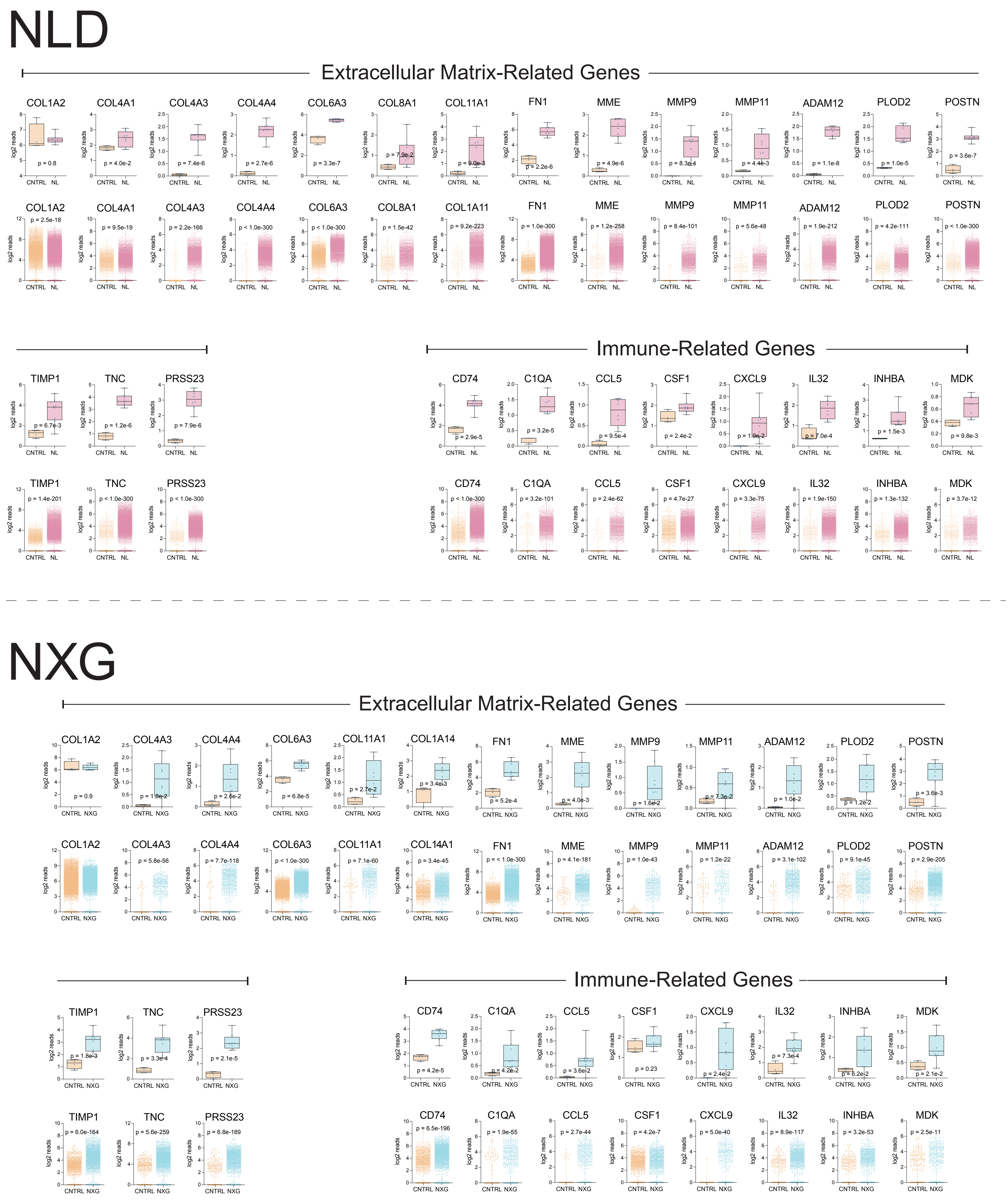

NLD
Extracellular Matrix-Related Genes
COL1A2 p = 0.8
COL4A1 p = 4.0e-2
COL4A3 p = 7.4e-6
COL4A4 p = 2.7e-6
COL6A3 p = 3.3e-7
COL8A1 p = 7.9e-2
COL11A1 p = 9.0e-3
FN1 p = 2.2e-6
MME p = 4.9e-6
MMP9 p = 8.3e-4
MMP11 p = 4.4e-3
ADAM12 p = 1.1e-8
PLOD2 p = 1.0e-5
POSTN p = 3.6e-7
COL1A2 p = 2.5e-18
COL4A1 p = 9.5e-19
COL4A3 p = 2.2e-166
COL4A4 p < 1.0e-300
COL6A3 p < 1.0e-300
COL8A1 p = 1.5e-42
COL1A11 p = 9.2e-223
FN1 p = 1.0e-300
MME p = 1.2e-258
MMP9 p = 8.4e-101
MMP11 p = 5.6e-48
ADAM12 p = 1.9e-212
PLOD2 p = 4.2e-111
POSTN p < 1.0e-300
TIMP1 p = 6.7e-3
TNC p = 1.2e-6
PRSS23 p = 7.9e-6
TIMP1 p = 1.4e-201
TNC p < 1.0e-300
PRSS23 p < 1.0e-300
Immune-Related Genes
CD74 p = 2.9e-5
C1QA p = 3.2e-5
CCL5 p = 9.5e-4
CSF1 p = 2.4e-2
CXCL9 p = 1.9e-2
IL32 p = 7.0e-4
INHBA p = 1.5e-3
MDK p = 9.8e-3
CD74 p < 1.0e-300
C1QA p = 3.2e-101
CCL5 p = 2.4e-62
CSF1 p = 4.7e-27
CXCL9 p = 3.3e-75
IL32 p = 1.9e-150
INHBA p = 1.3e-132
MDK p = 3.7e-12
log2 reads
CNTRL NL
NXG
Extracellular Matrix-Related Genes
COL1A2 p = 0.9
COL4A3 p = 1.8e-2
COL4A4 p = 2.6e-2
COL6A3 p = 6.8e-5
COL11A1 p = 2.7e-2
COL1A14 p = 3.4e-3
FN1 p = 5.2e-4
MME p = 4.0e-3
MMP9 p = 1.6e-2
MMP11 p = 7.3e-2
ADAM12 p = 1.0e-2
PLOD2 p = 1.2e-2
POSTN p = 3.6e-3
COL1A2
COL4A3 p = 5.8e-56
COL4A4 p = 7.7e-118
COL6A3 p < 1.0e-300
COL11A1 p = 7.1e-60
COL14A1 p = 3.4e-45
FN1 p = < 1.0e-300
MME p = 4.1e-181
MMP9 p = 1.0e-43
MMP11 p = 1.2e-22
ADAM12 p = 3.1e-102
PLOD2 p = 9.1e-45
POSTN p = 2.9e-205
TIMP1 p = 1.8e-3
TNC p = 3.3e-4
PRSS23 p = 2.1e-5
TIMP1 p = 6.0e-164
TNC p = 5.6e-259
PRSS23 p = 6.8e-189
Immune-Related Genes
CD74 p = 4.2e-5
C1QA p = 4.2e-2
CCL5 p = 3.6e-2
CSF1 p = 0.23
CXCL9 p = 2.4e-2
IL32 p = 7.3e-4
INHBA p = 6.2e-2
MDK p = 2.1e-2
CD74 p = 6.5e-196
C1QA p = 1.9e-55
CCL5 p = 2.7e-44
CSF1 p = 4.2e-7
CXCL9 p = 5.0e-40
IL32 p = 8.9e-117
INHBA p = 3.2e-53
MDK p = 2.5e-11
log2 reads
CNTRL NXG

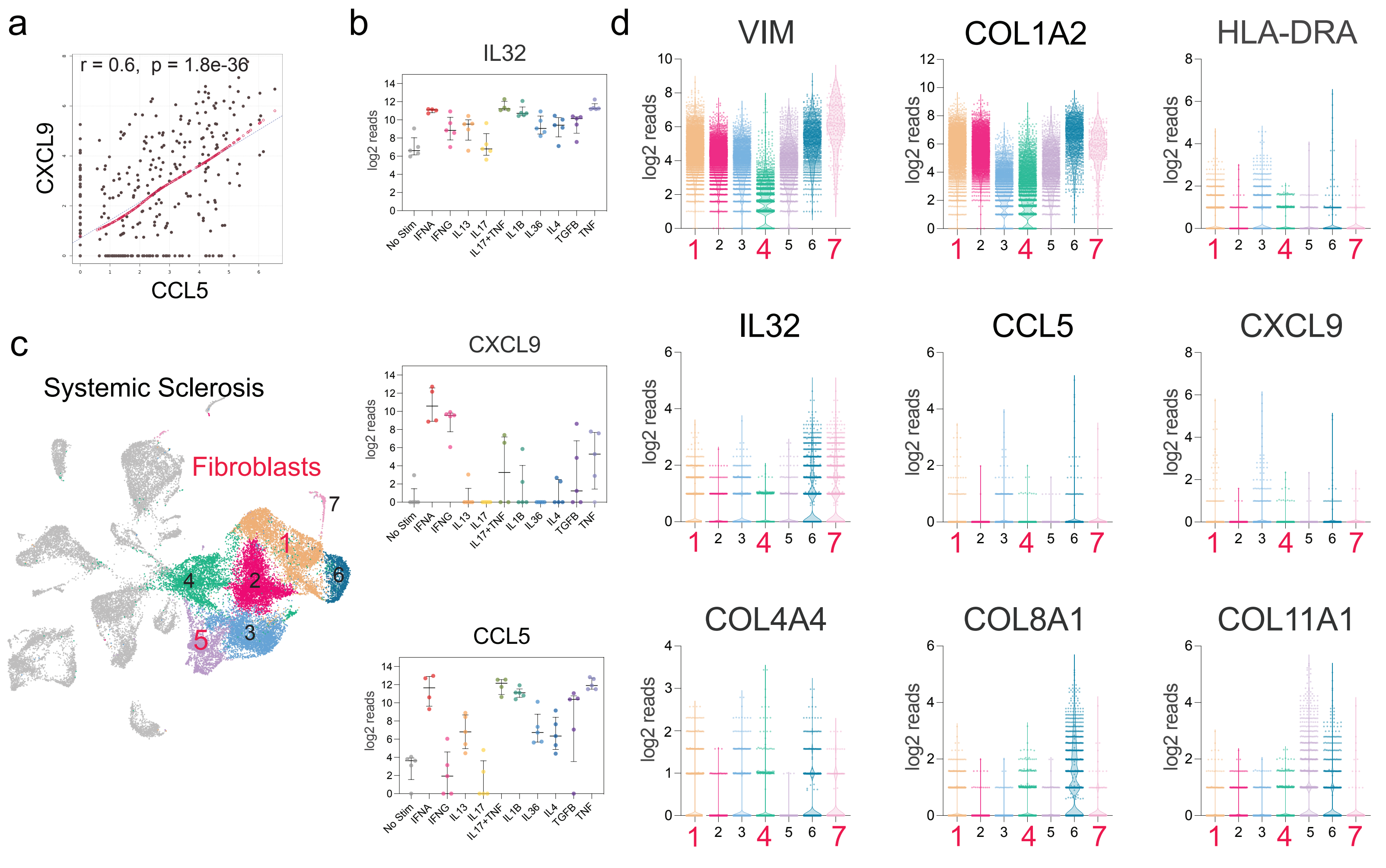

a
r = 0.6, p = 1.8e-36
CXCL9
CCL5
b
IL32
log2 reads
No Stim
IFNA
IFNG
IL13
IL17
IL17+TNF
IL1B
IL36
IL4
TGFB
TNF
CXCL9
CCL5
c
Systemic Sclerosis
Fibroblasts
1
2
3
4
5
6
7
d
VIM
COL1A2
HLA-DRA
IL32
CCL5
CXCL9
COL4A4
COL8A1
COL11A1
log2 reads

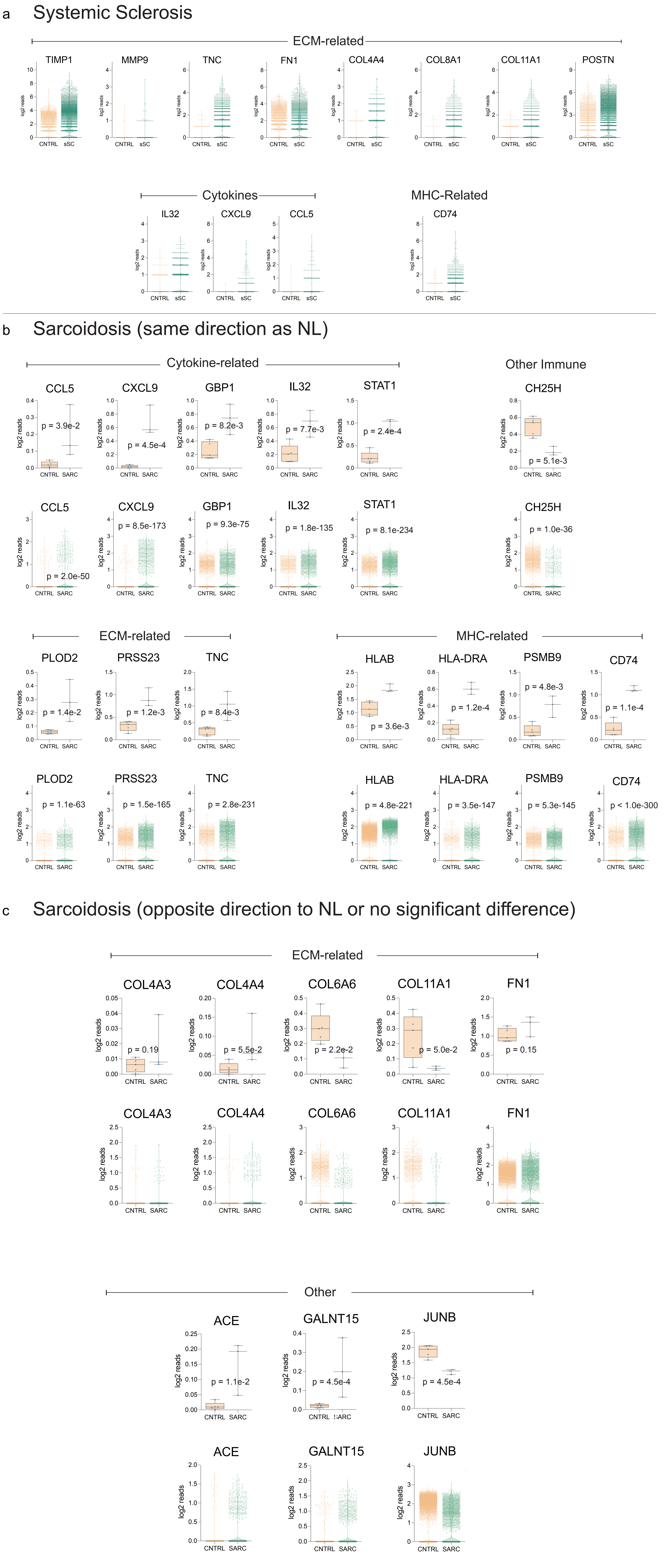
a
Systemic Sclerosis
ECM-related
TIMP1
MMP9
TNC
FN1
COL4A4
COL8A1
COL11A1
POSTN
Cytokines
IL32
CXCL9
CCL5
MHC-Related
CD74
CNTRL
sSC
log2 reads
b
Sarcoidosis (same direction as NL)
Cytokine-related
CCL5
p = 3.9e-2
CXCL9
p = 4.5e-4
GBP1
p = 8.2e-3
IL32
p = 7.7e-3
STAT1
p = 2.4e-4
Other Immune
CH25H
p = 5.1e-3
CCL5
p = 2.0e-50
CXCL9
p = 8.5e-173
GBP1
p = 9.3e-75
IL32
p = 1.8e-135
STAT1
p = 8.1e-234
CH25H
p = 1.0e-36
ECM-related
PLOD2
p = 1.4e-2
PRSS23
p = 1.2e-3
TNC
p = 8.4e-3
MHC-related
HLAB
p = 3.6e-3
HLA-DRA
p = 1.2e-4
PSMB9
p = 4.8e-3
CD74
p = 1.1e-4
PLOD2
p = 1.1e-63
PRSS23
p = 1.5e-165
TNC
p = 2.8e-231
HLAB
p = 4.8e-221
HLA-DRA
p = 3.5e-147
PSMB9
p = 5.3e-145
CD74
p < 1.0e-300
CNTRL
SARC
log2 reads
c
Sarcoidosis (opposite direction to NL or no significant difference)
ECM-related
COL4A3
p = 0.19
COL4A4
p = 5.5e-2
COL6A6
p = 2.2e-2
COL11A1
p = 5.0e-2
FN1
p = 0.15
Other
ACE
p = 1.1e-2
GALNT15
p = 4.5e-4
JUNB
p = 4.5e-4

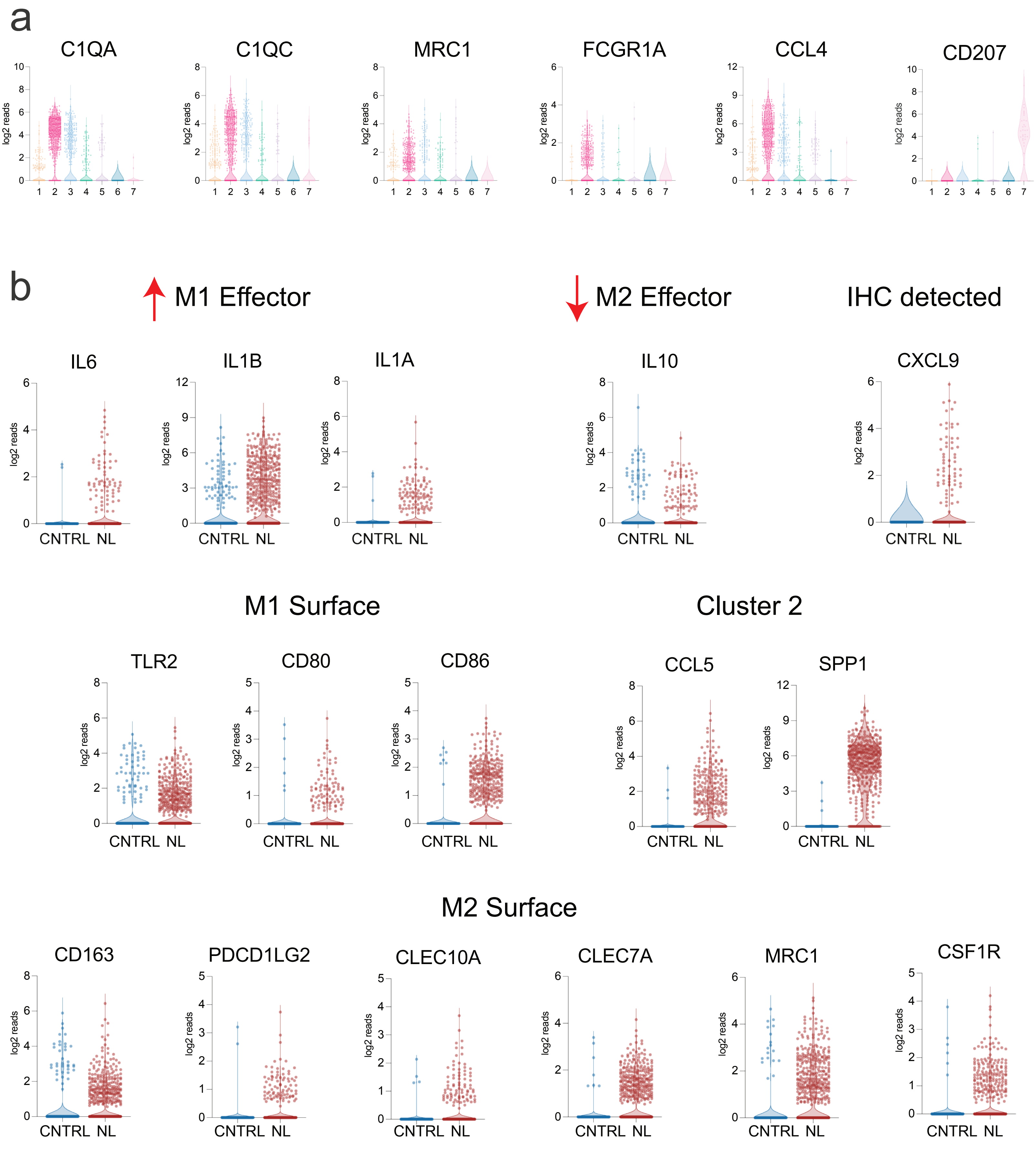
a
C1QA
C1QC
MRC1
FCGR1A
CCL4
CD207
log2 reads
b
M1 Effector
M2 Effector
IHC detected
IL6
IL1B
IL1A
IL10
CXCL9
CNTRL NL
M1 Surface
Cluster 2
TLR2
CD80
CD86
CCL5
SPP1
M2 Surface
CD163
PDCD1LG2
CLEC10A
CLEC7A
MRC1
CSF1R

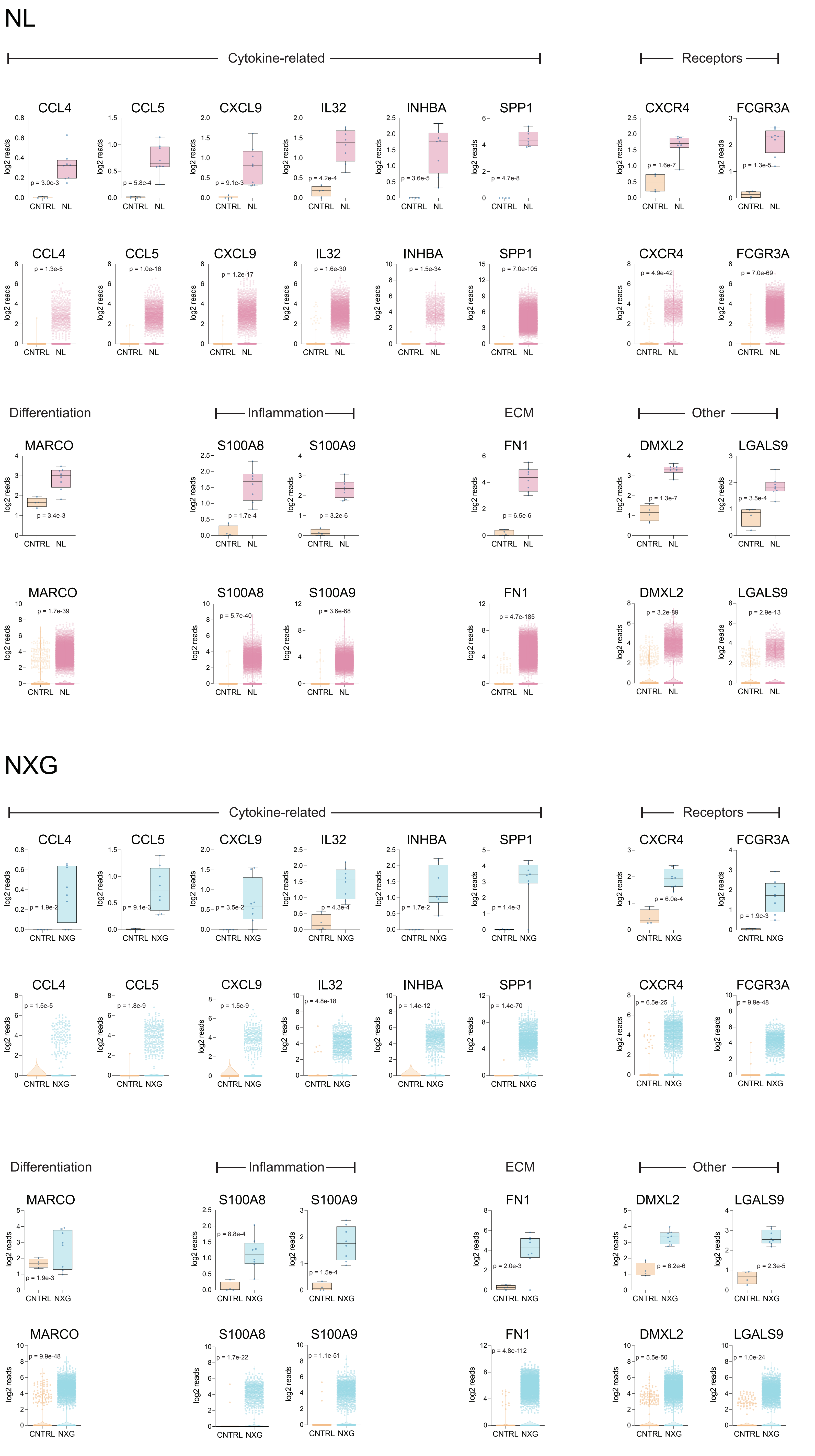
NL
Cytokine-related
Receptors
CCL4
p = 3.0e-3
CCL5
p = 5.8e-4
CXCL9
p = 9.1e-3
IL32
p = 4.2e-4
INHBA
p = 3.6e-5
SPP1
p = 4.7e-8
CXCR4
p = 1.6e-7
FCGR3A
p = 1.3e-5
CCL4
p = 1.3e-5
CCL5
p = 1.0e-16
CXCL9
p = 1.2e-17
IL32
p = 1.6e-30
INHBA
p = 1.5e-34
SPP1
p = 7.0e-105
CXCR4
p = 4.9e-42
FCGR3A
p = 7.0e-69
Differentiation
Inflammation
ECM
Other
MARCO
p = 3.4e-3
S100A8
p = 1.7e-4
S100A9
p = 3.2e-6
FN1
p = 6.5e-6
DMXL2
p = 1.3e-7
LGALS9
p = 3.5e-4
MARCO
p = 1.7e-39
S100A8
p = 5.7e-40
S100A9
p = 3.6e-68
FN1
p = 4.7e-185
DMXL2
p = 3.2e-89
LGALS9
p = 2.9e-13
log2 reads
CNTRL
NL
NXG
Cytokine-related
Receptors
CCL4
p = 1.9e-2
CCL5
p = 9.1e-3
CXCL9
p = 3.5e-2
IL32
p = 4.3e-4
INHBA
p = 1.7e-2
SPP1
p = 1.4e-3
CXCR4
p = 6.0e-4
FCGR3A
p = 1.9e-3
CCL4
p = 1.5e-5
CCL5
p = 1.8e-9
CXCL9
p = 1.5e-9
IL32
p = 4.8e-18
INHBA
p = 1.4e-12
SPP1
p = 1.4e-70
CXCR4
p = 6.5e-25
FCGR3A
p = 9.9e-48
Differentiation
Inflammation
ECM
Other
MARCO
p = 1.9e-3
S100A8
p = 8.8e-4
S100A9
p = 1.5e-4
FN1
p = 2.0e-3
DMXL2
p = 6.2e-6
LGALS9
p = 2.3e-5
MARCO
p = 9.9e-48
S100A8
p = 1.7e-22
S100A9
p = 1.1e-51
FN1
p = 4.8e-112
DMXL2
p = 5.5e-50
LGALS9
p = 1.0e-24
log2 reads
CNTRL
NXG

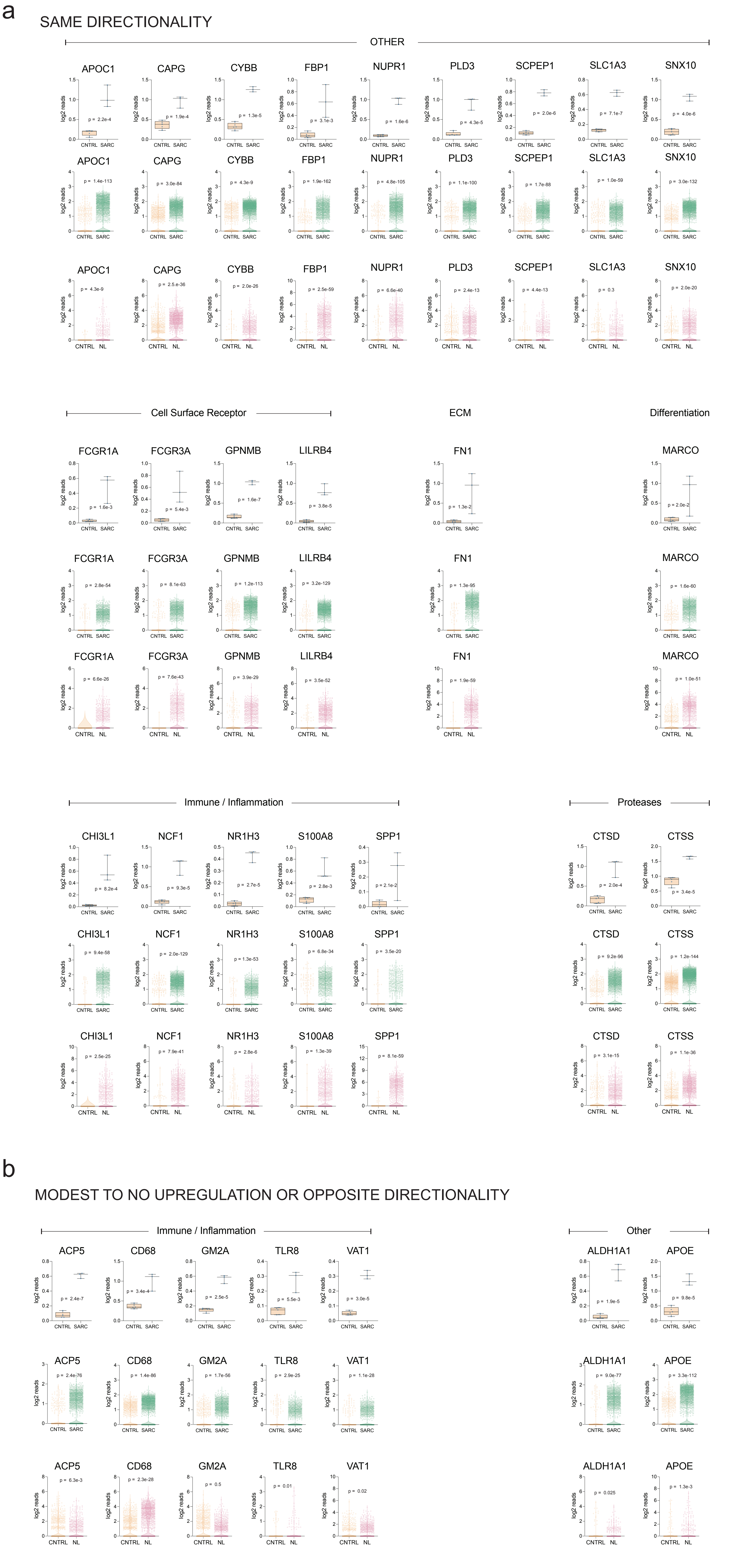
a
SAME DIRECTIONALITY
OTHER
APOC1
CAPG
CYBB
FBP1
NUPR1
PLD3
SCPEP1
SLC1A3
SNX10
p = 2.2e-4
p = 1.9e-4
p = 1.3e-5
p = 3.1e-3
p = 1.6e-6
p = 4.3e-5
p = 2.0e-6
p = 7.1e-7
p = 4.0e-6
p = 1.4e-113
p = 3.0e-84
p = 4.3e-9
p = 1.9e-162
p = 4.8e-105
p = 1.1e-100
p = 1.7e-88
p = 1.0e-59
p = 3.0e-132
p = 4.3e-9
p = 2.5e-36
p = 2.0e-26
p = 2.5e-59
p = 6.6e-40
p = 2.4e-13
p = 4.4e-13
p = 0.3
p = 2.0e-20
Cell Surface Receptor
ECM
Differentiation
FCGR1A
FCGR3A
GPNMB
LILRB4
FN1
MARCO
p = 1.6e-3
p = 5.4e-3
p = 1.6e-7
p = 3.8e-5
p = 1.3e-2
p = 2.0e-2
p = 2.8e-54
p = 8.1e-63
p = 1.2e-113
p = 3.2e-129
p = 1.3e-95
p = 1.6e-60
p = 6.6e-26
p = 7.6e-43
p = 3.9e-29
p = 3.5e-52
p = 1.9e-59
p = 1.0e-51
Immune / Inflammation
Proteases
CHI3L1
NCF1
NR1H3
S100A8
SPP1
CTSD
CTSS
p = 8.2e-4
p = 9.3e-5
p = 2.7e-5
p = 2.8e-3
p = 2.1e-2
p = 2.0e-4
p = 3.4e-5
p = 9.4e-58
p = 2.0e-129
p = 1.3e-53
p = 6.8e-34
p = 3.5e-20
p = 9.2e-96
p = 1.2e-144
p = 2.5e-25
p = 7.9e-41
p = 2.8e-6
p = 1.3e-39
p = 8.1e-59
p = 3.1e-15
p = 1.1e-36
b
MODEST TO NO UPREGULATION OR OPPOSITE DIRECTIONALITY
Immune / Inflammation
Other
ACP5
CD68
GM2A
TLR8
VAT1
ALDH1A1
APOE
p = 2.4e-7
p = 3.4e-4
p = 2.5e-5
p = 5.5e-3
p = 3.0e-5
p = 1.9e-5
p = 9.8e-5
p = 2.4e-76
p = 1.4e-86
p = 1.7e-56
p = 2.9e-25
p = 1.1e-28
p = 9.0e-77
p = 3.3e-112
p = 6.3e-3
p = 2.3e-28
p = 0.5
p = 0.01
p = 0.02
p = 0.025
p = 1.3e-3
log2 reads
CNTRL
SARC
NL

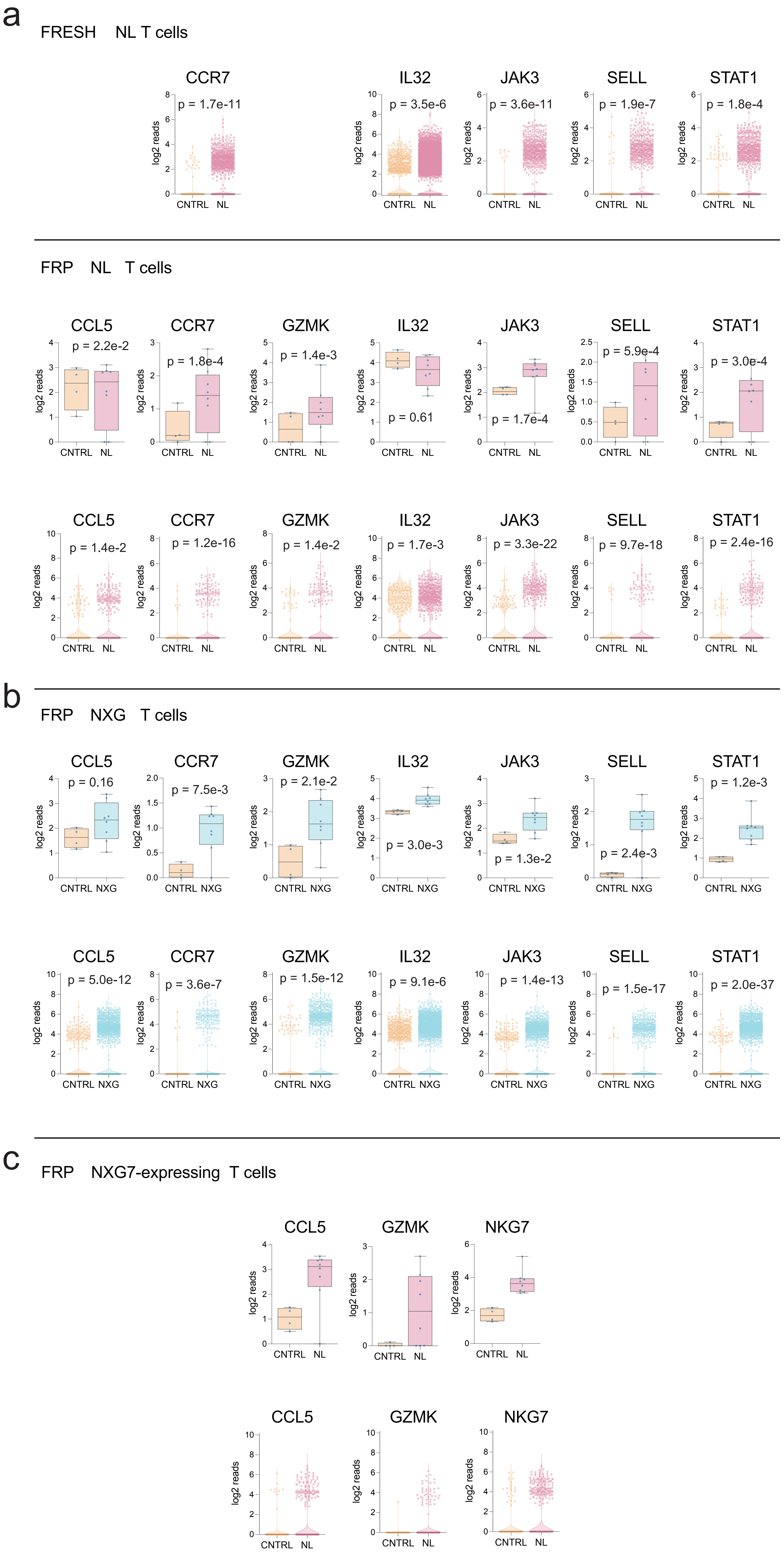

a
FRESH NL T cells
CCR7
p = 1.7e-11
IL32
p = 3.5e-6
JAK3
p = 3.6e-11
SELL
p = 1.9e-7
STAT1
p = 1.8e-4
log2 reads
CNTRL NL
FRP NL T cells
CCL5
p = 2.2e-2
CCR7
p = 1.8e-4
GZMK
p = 1.4e-3
IL32
p = 0.61
JAK3
p = 1.7e-4
SELL
p = 5.9e-4
STAT1
p = 3.0e-4
CCL5
p = 1.4e-2
CCR7
p = 1.2e-16
GZMK
p = 1.4e-2
IL32
p = 1.7e-3
JAK3
p = 3.3e-22
SELL
p = 9.7e-18
STAT1
p = 2.4e-16
b
FRP NXG T cells
CCL5
p = 0.16
CCR7
p = 7.5e-3
GZMK
p = 2.1e-2
IL32
p = 3.0e-3
JAK3
p = 1.3e-2
SELL
p = 2.4e-3
STAT1
p = 1.2e-3
CNTRL NXG
CCL5
p = 5.0e-12
CCR7
p = 3.6e-7
GZMK
p = 1.5e-12
IL32
p = 9.1e-6
JAK3
p = 1.4e-13
SELL
p = 1.5e-17
STAT1
p = 2.0e-37
c
FRP NXG7-expressing T cells
CCL5
GZMK
NKG7
CNTRL NL
CCL5
GZMK
NKG7

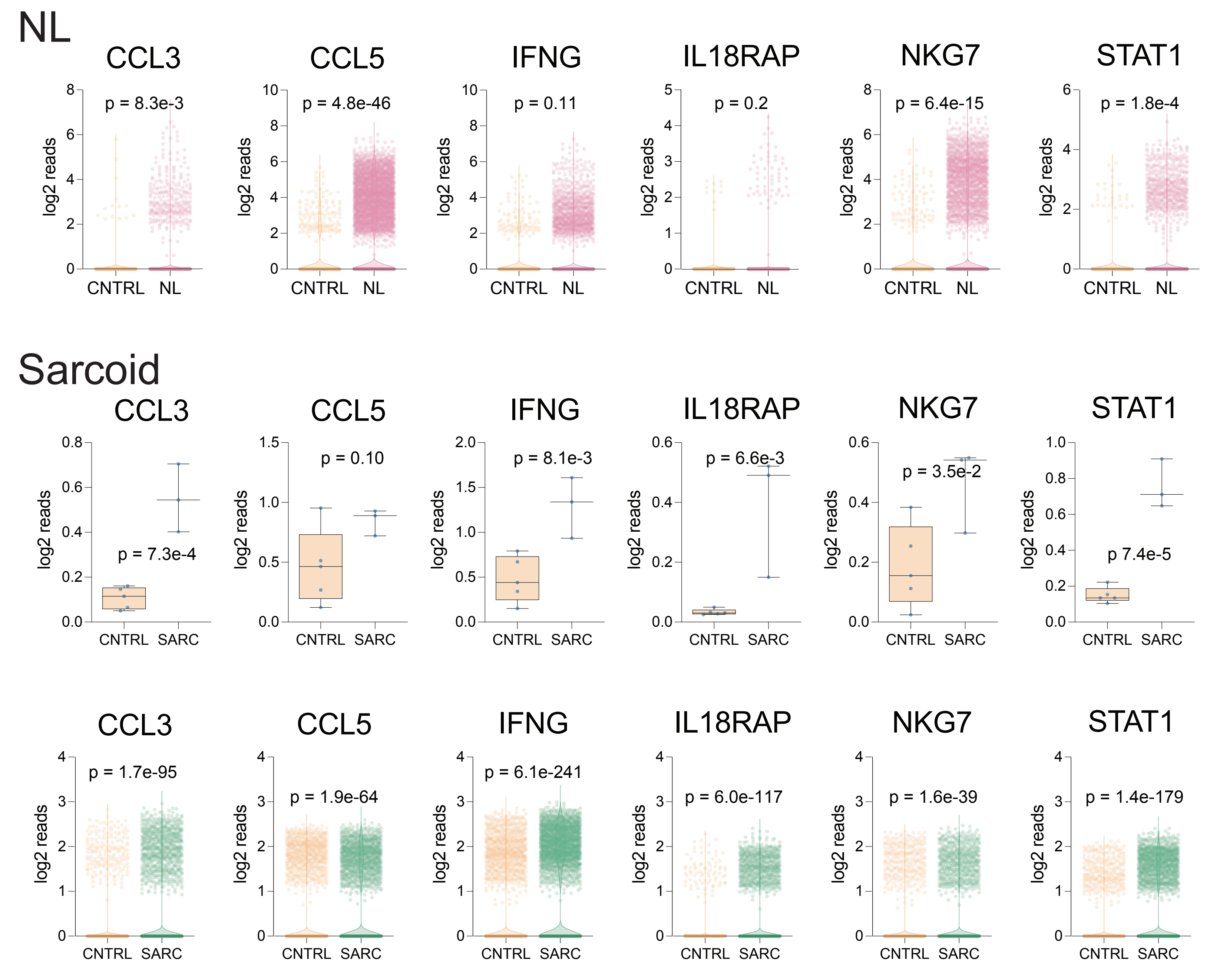
NL
CCL3
p = 8.3e-3
CCL5
p = 4.8e-46
IFNG
p = 0.11
IL18RAP
p = 0.2
NKG7
p = 6.4e-15
STAT1
p = 1.8e-4
log2 reads
CNTRL
NL
Sarcoid
CCL3
p = 7.3e-4
CCL5
p = 0.10
IFNG
p = 8.1e-3
IL18RAP
p = 6.6e-3
NKG7
p = 3.5e-2
STAT1
p 7.4e-5
log2 reads
CNTRL
SARC
CCL3
p = 1.7e-95
CCL5
p = 1.9e-64
IFNG
p = 6.1e-241
IL18RAP
p = 6.0e-117
NKG7
p = 1.6e-39
STAT1
p = 1.4e-179
log2 reads
CNTRL
SARC

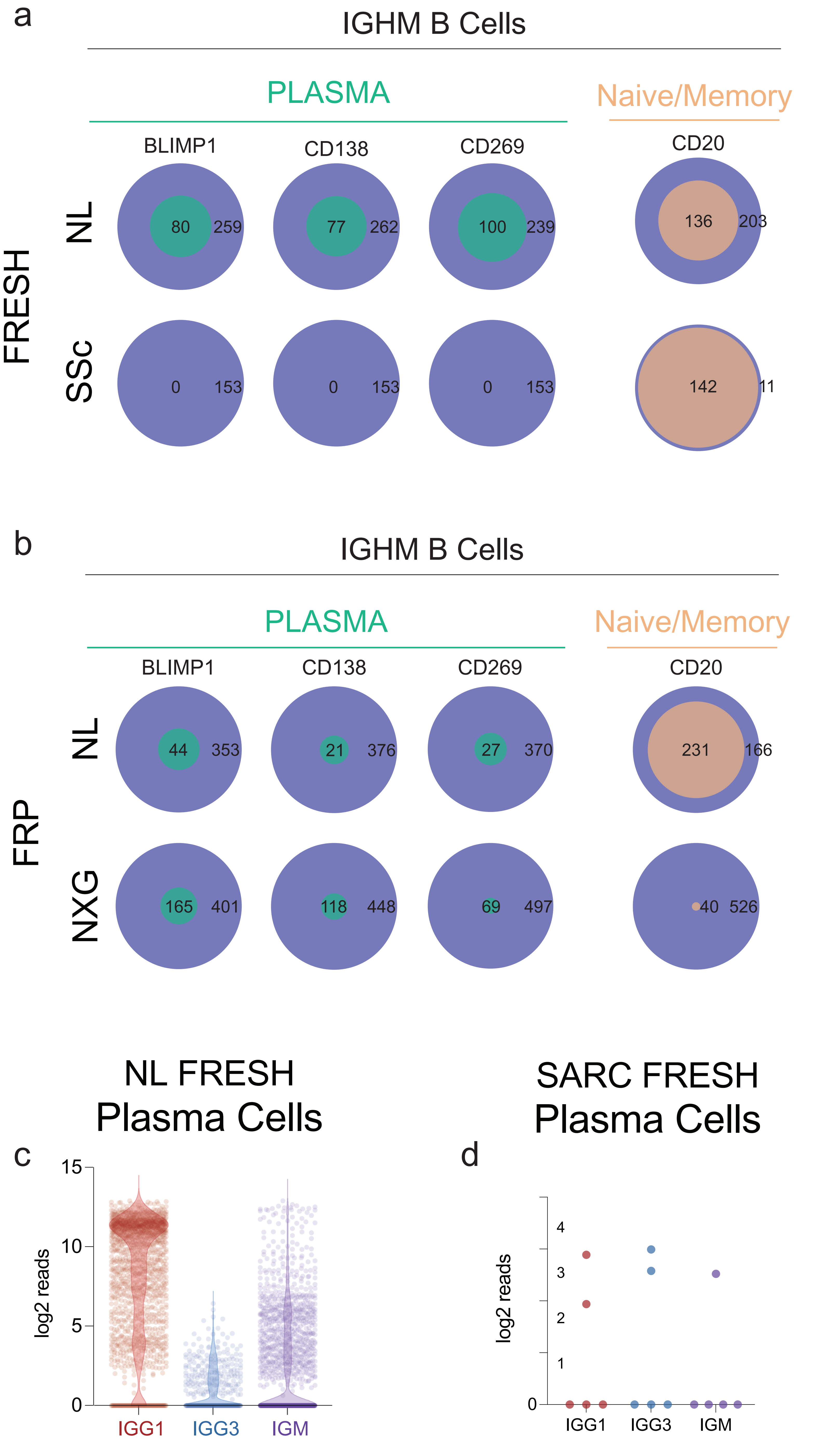
a
IGHM B Cells
PLASMA
Naive/Memory
BLIMP1
CD138
CD269
CD20
FRESH
NL
80
259
77
262
100
239
136
203
SSc
0
153
0
153
0
153
142
11
b
IGHM B Cells
PLASMA
Naive/Memory
BLIMP1
CD138
CD269
CD20
FRP
NL
44
353
21
376
27
370
231
166
NXG
165
401
118
448
69
497
40
526
NL FRESH
Plasma Cells
c
log2 reads
15
10
5
0
IGG1
IGG3
IGM
SARC FRESH
Plasma Cells
d
log2 reads
4
3
2
1
0
IGG1
IGG3
IGM

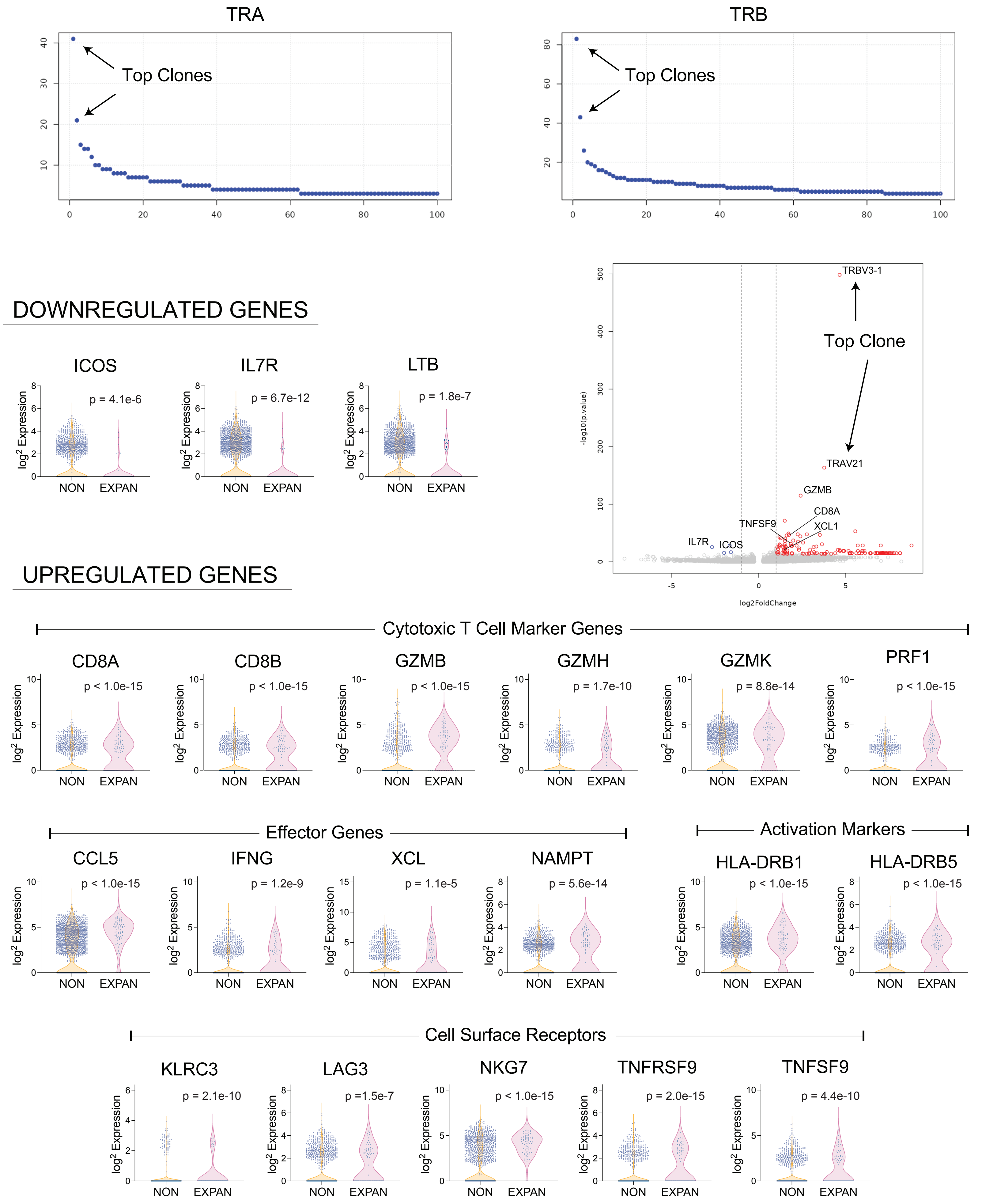
TRA
TRB
Top Clones
Top Clones
DOWNREGULATED GENES
ICOS
p = 4.1e-6
IL7R
p = 6.7e-12
LTB
p = 1.8e-7
log2 Expression
NON
EXPAN
TRBV3-1
Top Clone
TRAV21
GZMB
CD8A
XCL1
TNFSF9
IL7R
ICOS
-log10(p.value)
log2FoldChange
UPREGULATED GENES
Cytotoxic T Cell Marker Genes
CD8A
p < 1.0e-15
CD8B
p < 1.0e-15
GZMB
p < 1.0e-15
GZMH
p = 1.7e-10
GZMK
p = 8.8e-14
PRF1
p < 1.0e-15
Effector Genes
CCL5
p < 1.0e-15
IFNG
p = 1.2e-9
XCL
p = 1.1e-5
NAMPT
p = 5.6e-14
Activation Markers
HLA-DRB1
p < 1.0e-15
HLA-DRB5
p < 1.0e-15
Cell Surface Receptors
KLRC3
p = 2.1e-10
LAG3
p =1.5e-7
NKG7
p < 1.0e-15
TNFRSF9
p = 2.0e-15
TNFSF9
p = 4.4e-10
